# Comparison of anthropometric and body composition indices in the identification of metabolic risk factors

**DOI:** 10.1038/s41598-021-89422-x

**Published:** 2021-05-11

**Authors:** Bum Ju Lee, Mi Hong Yim

**Affiliations:** Future Medicine Division, Korea Institute of Oriental Medicine, Deajeon, 305-811 Republic of Korea

**Keywords:** Diseases, Health care, Medical research, Risk factors

## Abstract

Whether anthropometric or body composition indices are better indicators of metabolic risk remains unclear. The objectives of this study were to compare the association of metabolic risk factors with anthropometric and body composition indices and to identify the better indicators for risk factors in a large-scale Korean population. In this cross-sectional study, the associations of body mass index (BMI), waist circumference (WC), and waist-to-height ratio (WHtR) as anthropometric indices and trunk fat mass (TFM), percent trunk fat mass (%TFM), whole-body total fat mass (WBTFM), and percent whole-body total fat mass (%WBTFM) as body composition indices with metabolic risk factors were compared by complex-samples multiple logistic regression models based on complex-sample survey data. In men, WHtR, BMI, and TFM were similarly associated with hypertension. Diabetes, hyperlipidemia, and hypo-high-density lipoprotein (HDL) cholesterolemia tended to be more strongly associated with WHtR and WC than body composition indices. Hypertriglyceridemia and hypercholesterolemia were more strongly associated with WHtR and %TFM than other indices. In women, hypertension tended to be more strongly associated with WHtR than other indices. TFM, %TFM, and WHtR were similarly associated with hyperlipidemia. Diabetes and hypo-HDL cholesterolemia were more strongly associated with WHtR and WC than body composition indices. Hypertriglyceridemia and hypercholesterolemia were more strongly associated with WHtR and %TFM than other indices. Among six metabolic risk factors, the validity and utility of the anthropometric indices in identifying risk factors tended to be similar to or better than those of the body composition indices, except for hypertension and hypercholesterolemia in men and hyperlipidemia and hypercholesterolemia in women.

## Introduction

Obesity is one of the most serious health problems in most countries^1,2^. Obesity and adiposity increase the risk of serious and common diseases, such as cancer, hypertension, sleep apnea, diabetes, metabolic abnormalities, and cardiovascular diseases, and can lead to disability and premature death^1,3,4^. Therefore, obesity is not a matter of beauty but a matter of health and serious diseases worldwide^1,2^. For a long time, many studies have been conducted to discover useful indicators of obesity and adiposity to identify serious chronic diseases, to reveal associations between obesity indicators and related diseases, and to find obesity-related genes by genome-wide association^2,5^. There are two methods to measure indicators or indices of obesity and adiposity: anthropometric indices, such as body mass index (BMI), waist circumference (WC), waist-to-height ratio (WHtR), and waist-to-hip ratio (WHR), can be determined by simple measurements, and body composition indices, such as body fat mass and lean mass, percent fat mas, and trunk fat mass (TFM), can be determined by dual X-ray absorptiometry (DXA), magnetic resonance imaging (MRI), and bioelectrical impedance (BIA).

Most studies on screening for or the identification of various chronic diseases using these indices have two main issues. First, until now, although many studies have been performed on the association of various chronic diseases with various anthropometric and body composition indices, the best indicator of these diseases remains unclear because the best indicator among various indices differs according to the specific disease^6^, age and sex^7^, and ethnic group and country^6,8,9^. The second important issue is that the superiority of body composition or anthropometric indices in identifying risk factors for cardiovascular disease (CVD) and metabolic abnormalities remains unclear, and the two methods of measurement are very different in cost, time, availability, technical experience, ease or difficulty of measurement, and large-scale research^10,11^. Furthermore, studies on this issue are very rare. Although several previous studies have compared anthropometric and body composition indices in several chronic diseases, the studies were limited by ethnic group, country, age, sex, or the small number of subjects.

This study focused on the second issue regarding the comparison of body composition and anthropometric indices in screening for or identifying risk factors for CVD and metabolic abnormalities in a large-scale population. Therefore, the objectives of the present study were to compare the associations of metabolic risk factors with anthropometric and body composition indices and to identify which method is superior in screening for or identifying risk factors. Furthermore, we aimed to identify the best indicators of metabolic risk factors in the Korean population.

## Results

Table 1 presents the demographic characteristics of the subjects in this study according to men, women, and both men and women. The mean ± standard error of the subjects’ age was 43.73 ± 0.33 in men and 45.47 ± 0.33 in women. Most of variables were significantly associated with gender, except for cholesterol, sleep duration, region, town, and income.

**Table 1:** Basic characteristics of the subjects in this study.

Variables	Men and women	*P *value	Men	Women
Number of subjects	10,790		4433	6,357
Age (years)	44.65 ± 0.28	< .001	43.73 ± 0.33	45.47 ± 0.33
BMI (kg/m^2^)	23.65 ± 0.05	< .001	24.02 ± 0.06	23.32 ± 0.06
Waist circumference (cm)	80.73 ± 0.15	< .001	83.84 ± 0.19	77.95 ± 0.20
Waist-to-height ratio	0.49 ± 0.001	.001	0.49 ± 0.001	0.50 ± 0.001
SBP (mmHg)	118.52 ± 0.27	< .001	121.46 ± 0.31	115.89 ± 0.33
DBP (mmHg)	77.43 ± 0.18	< .001	80.75 ± 0.23	74.46 ± 0.20
Pulse rate (beats per 15 s)	17.75 ± 0.04	< .001	17.57 ± 0.05	17.92 ± 0.05
Hemoglobin (mg/dl)	14.08 ± 0.02	< .001	15.33 ± 0.02	12.97 ± 0.02
Cholesterol (mg/dl)	187.20 ± 0.47	.813	187.31 ± 0.67	187.10 ± 0.60
Triglyceride (mg/dl)	131.73 ± 1.35	< .001	155.64 ± 2.45	110.40 ± 1.23
Glucose (mg/dl)	95.98 ± 0.28	< .001	97.76 ± 0.44	94.39 ± 0.30
AST (IU/L)	22.24 ± 0.15	< .001	24.92 ± 0.27	19.86 ± 0.12
ALT (IU/L)	21.97 ± 0.21	< .001	27.25 ± 0.36	17.26 ± 0.19
Creatinine (mg/dl)	0.82 ± 0.003	< .001	0.96 ± 0.004	0.70 ± 0.002
Trunk fat mass (kg)	9.17 ± 0.06	< .001	8.58 ± 0.08	9.70 ± 0.07
Percent trunk fat mass (%)	29.06 ± 0.15	< .001	24.42 ± 0.17	33.20 ± 0.17
Whole-body total fat mass (kg)	17.60 ± 0.10	< .001	15.73 ± 0.13	19.28 ± 0.11
Percent whole-body total fat mass (%)	27.99 ± 0.14	< .001	22.17 ± 0.14	33.19 ± 0.13
Sleep, mean duration (hours)	6.88 ± 0.02	.517	6.89 ± 0.02	6.87 ± 0.02
**Region (city)**		.716		
Seoul	21.17 (0.94)		21.58 (1.13)	20.8 (0.94)
Busan	7.85 (0.75)		7.89 (0.77)	7.82 (0.85)
Daegu	5.13 (0.71)		5.15 (0.76)	5.11 (0.71)
Incheon	5.22 (0.55)		5.30 (0.58)	5.15 (0.57)
Gwangju	2.24 (0.64)		2.27 (0.71)	2.21 (0.61)
Daejeon	3.28 (0.65)		3.18 (0.68)	3.36 (0.69)
Ulsan	2.34 (0.69)		2.11 (0.64)	2.55 (0.76)
Gyeonggi-do	22.92 (0.97)		22.85 (1.12)	22.98 (1.04)
Gangwon-do	2.33 (0.45)		2.34 (0.45)	2.32 (0.50)
Chungcheongbuk-do	3.35 (0.59)		3.36 (0.61)	3.34 (0.62)
Chungcheongnam-do	4.29 (0.65)		3.95 (0.66)	4.59 (0.68)
Jeollabuk-do	3.02 (0.44)		2.98 (0.47)	3.06 (0.47)
Jeollanam-do	3.04 (0.47)		2.8 (0.48)	3.24 (0.51)
Gyeongsangbuk-do	5.70 (0.68)		5.58 (0.72)	5.79 (0.74)
Gyeongsangnam-do	6.25 (0.84)		6.83 (0.97)	5.73 (0.78)
Jeju-do	1.89 (0.80)		1.82 (0.67)	1.95 (0.96)
**Town**		.730		
Dong (city)	80.49 (1.78)		80.62 (1.83)	80.37 (1.81)
Eup, Myeon (rural)	19.51 (1.78)		19.38 (1.83)	19.63 (1.81)
**Income**		.169		
1st quartile (low)	26.12 (0.75)		25.78 (0.96)	26.42 (0.85)
2nd quartile (lower-middle)	25.80 (0.67)		26.27 (0.89)	25.37 (0.74)
3rd quartile (upper-middle)	24.19 (0.60)		23.33 (0.76)	24.97 (0.73)
4th quartile (high)	23.89 (0.78)		24.62 (0.98)	23.24 (0.84)
**Education**		< .001		
Elementary school or less	19.12 (0.67)		11.98 (0.61)	25.50 (0.89)
Middle school	9.88 (0.35)		10.32 (0.52)	9.50 (0.44)
High school	38.81 (0.78)		40.35 (1.06)	37.45 (0.96)
University or higher	32.18 (0.88)		37.36 (1.14)	27.56 (0.89)
**Occupation**		< .001		
Managers, professionals and related workers	13.85 (0.52)		17.91 (0.79)	10.23 (0.51)
Clerks	8.85 (0.35)		11.60 (0.59)	6.39 (0.36)
Service workers and sale workers	13.74 (0.50)		12.84 (0.66)	14.55 (0.62)
Skilled agricultural, forestry and fishery workers	6.55 (0.74)		7.71 (0.89)	5.51 (0.66)
Craft, plant, machine operators and assemblers	10.69 (0.43)		20.09 (0.82)	2.30 (0.23)
Elementary occupations	7.85 (0.35)		7.25 (0.49)	8.40 (0.45)
Unemployed	38.47 (0.69)		22.61 (0.91)	52.62 (0.86)
**Drinking**		< .001		
Not at all for the past one year	23.62 (0.58)		13.01 (0.65)	33.08 (0.84)
Less than once a month	18.68 (0.48)		9.68 (0.61)	26.72 (0.68)
Once a month	10.55 (0.35)		9.23 (0.55)	11.73 (0.49)
2 to 4 times a month	24.77 (0.59)		30.70 (0.87)	19.48 (0.66)
2 or 3 times a week	15.09 (0.45)		24.23 (0.78)	6.94 (0.44)
4 or more times a week	7.28 (0.33)		13.14 (0.62)	2.05 (0.22)
**Smoking**		< .001		
Smoking	25.36 (0.56)		46.4 (0.99)	6.58 (0.42)
Quit smoking	19.13 (0.47)		33.40 (0.87)	6.39 (0.41)
Never smoked	55.52 (0.55)		20.2 (0.76)	87.03 (0.58)
**Stress**		< .001		
Extremely	4.72 (0.25)		4.10 (0.34)	5.26 (0.34)
Very	24.45 (0.53)		22.36 (0.79)	26.32 (0.72)
Slightly	57.74 (0.59)		59.5 (0.88)	56.18 (0.75)
Rarely	13.09 (0.39)		14.04 (0.59)	12.24 (0.48)
**Exercise**		< .001		
Not at all	65.52 (0.70)		55.8 (0.97)	74.19 (0.81)
Once a week	10.85 (0.42)		15.66 (0.74)	6.56 (0.39)
2 times a week	7.35 (0.32)		9.72 (0.54)	5.24 (0.34)
3 times a week	6.46 (0.31)		7.26 (0.45)	5.74 (0.41)
4 times a week	2.68 (0.22)		3.19 (0.33)	2.23 (0.26)
5 times a week	3.12 (0.22)		3.49 (0.34)	2.78 (0.25)
6 times a week	1.51 (0.15)		2.12 (0.27)	0.97 (0.14)
Every day	2.51 (0.21)		2.76 (0.30)	2.29 (0.28)

*BMI* body mass index, *SBP* systolic blood pressure, *DBP* diastolic blood pressure, *AST* aspartate aminotransferase, *ALT* alanine aminotransferase. Continuous variables are represented as the mean ± standard error (SE) from complex-samples general linear models, and categorical variables are represented as the percentage (SE) from Rao-Scott chi-square tests. All statistical analyses were conducted using weight, cluster and stratification parameters to consider complex-sample survey data.

Tables 2 and 3 show the association of anthropometric and body composition indices with metabolic risk factors in men and women. In men, hypertension was more strongly associated with WHtR than other indices on crude analysis (OR [95% confidence interval) = 2.20 [1.99–2.44]), but the metabolic risk factor was similarly associated with BMI, WHtR, and TFM in model 1, adjusted for age, drinking, and smoking (OR = 1.90 [1.71–2.10], OR = 1.85 [1.65–2.09], and OR = 1.92 [1.71–2.15], respectively), and model 2, adjusted for age, drinking, smoking, exercise, income, town, education, occupation, and stress (OR = 1.90 [1.70–2.12], OR = 1.87 [1.66–2.10], and OR = 1.91 [1.70–2.15], respectively). Hyperlipidemia was more strongly associated with WHtR than the other indices in the crude analysis. However, the association was slightly stronger or similar compared to the other indices in adjusted models 1 and 2. Additionally, WHtR tended to be more strongly associated with diabetes than other indices in all models, and WHtR and WC were similarly associated with diabetes in adjusted model 1 (OR = 1.75 [1.52–2.02] and OR = 1.72 [1.50–1.96]) and model 2 (OR = 1.77 [1.54–2.02] and OR = 1.76 [1.54–2.00]). Hypercholesterolemia was more strongly associated with WHtR (OR = 1.76 [1.57–1.97]) and %TFM (OR = 1.75 [1.56–1.95]) than other indices on crude analysis, but hypercholesterolemia was more associated with %TFM than WHtR in adjusted model 1 (OR = 1.72 [1.53–1.92] and OR = 1.64 [1.46–1.84]) and model 2 (OR = 1.72 [1.53–1.92] and OR = 1.66 [1.48–1.86]). Hypo-HDL cholesterolemia was more strongly associated with WHtR than other indices in all models, and the association of WHtR was slightly stronger than that of WC. Hypertriglyceridemia tended to be more strongly associated with WHtR in all models, and the association of WHtR was slightly stronger than that of %TFM. Overall, in men, metabolic risk factors were more closely associated with WHtR than other indices, except for BMI and TFM in hypertension and %TFM in hypercholesterolemia in adjusted models 1 and 2. Comparing all anthropometric and body composition indices, WHtR was more closely associated with hyperlipidemia, diabetes, hypo-HDL cholesterolemia, and hypertriglyceridemia than the other indices, although the difference in magnitude of the associations was small.

**Table 2:** Association of CVD with anthropometric and body composition indices in men.

CVD	Analysis	BMI	WC	WHtR	TFM	%TFM	WBTFM	%WBTFM
Hypertension	Crude	1.51 (1.39–1.65)***	1.83 (1.66–2.00)***	2.20 (1.99–2.44)***	1.59 (1.45–1.74)***	1.79 (1.61–1.98)***	1.39 (1.28–1.52)***	1.60 (1.45–1.77)***
	Model 1	1.90 (1.71–2.10)***	1.83 (1.64–2.05)***	1.85 (1.65–2.09)***	1.92 (1.71–2.15)***	1.83 (1.63–2.06)***	1.84 (1.64–2.06)***	1.73 (1.54–1.95)***
	Model 2	1.90 (1.70–2.12)***	1.84 (1.65–2.06)***	1.87 (1.66–2.10)***	1.91 (1.70–2.15)***	1.83 (1.61–2.07)***	1.83 (1.63–2.06)***	1.73 (1.53–1.96)***
Hyperlipidemia	Crude	1.54 (1.36–1.73)***	1.72 (1.50–1.96)***	1.84 (1.60–2.10)***	1.58 (1.42–1.77)***	1.72 (1.52–1.94)***	1.42 (1.28–1.59)***	1.51 (1.34–1.70)***
	Model 1	1.65 (1.46–1.86)***	1.68 (1.47–1.91)***	1.70 (1.48–1.95)***	1.66 (1.48–1.86)***	1.66 (1.46–1.89)***	1.56 (1.39–1.75)***	1.50 (1.33–1.70)***
	Model 2	1.61 (1.42–1.82)***	1.66 (1.45–1.89)***	1.70 (1.48–1.95)***	1.61 (1.43–1.81)***	1.62 (1.42–1.84)***	1.53 (1.36–1.72)***	1.48 (1.30–1.68)***
Diabetes	Crude	1.33 (1.17–1.51)***	1.72 (1.50–1.97)***	2.02 (1.75–2.33)***	1.40 (1.25–1.56)***	1.50 (1.32–1.70)***	1.19 (1.06–1.33)**	1.32 (1.16–1.50)***
	Model 1	1.56 (1.36–1.80)***	1.72 (1.50–1.96)***	1.75 (1.52–2.02)***	1.60 (1.41–1.82)***	1.49 (1.29–1.72)***	1.44 (1.26–1.64)***	1.34 (1.16–1.55)***
	Model 2	1.61 (1.41–1.85)***	1.76 (1.54–2.00)***	1.77 (1.54–2.02)***	1.67 (1.47–1.90)***	1.53 (1.33–1.77)***	1.50 (1.31–1.71)***	1.38 (1.19–1.60)***
Hypercholesterolemia	Crude	1.46 (1.31–1.63)***	1.60 (1.43–1.80)***	1.76 (1.57–1.97)***	1.56 (1.41–1.73)***	1.75 (1.56–1.95)***	1.41 (1.27–1.56)***	1.57 (1.41–1.75)***
	Model 1	1.50 (1.35–1.67)***	1.54 (1.38–1.73)***	1.64 (1.46–1.84)***	1.60 (1.44–1.77)***	1.72 (1.53–1.92)***	1.49 (1.35–1.66)***	1.57 (1.41–1.75)***
	Model 2	1.49 (1.35–1.66)***	1.55 (1.39–1.73)***	1.66 (1.48–1.86)***	1.60 (1.44–1.76)***	1.72 (1.53–1.92)***	1.49 (1.35–1.65)***	1.58 (1.42–1.76)***
Hypo-HDL cholesterolemia	Crude	1.52 (1.39–1.66)***	1.61 (1.47–1.76)***	1.65 (1.52–1.80)***	1.54 (1.41–1.68)***	1.59 (1.47–1.73)***	1.48 (1.36–1.61)***	1.55 (1.43–1.68)***
	Model 1	1.60 (1.46–1.75)***	1.65 (1.51–1.80)***	1.68 (1.54–1.85)***	1.61 (1.48–1.75)***	1.64 (1.51–1.77)***	1.59 (1.45–1.73)***	1.59 (1.47–1.72)***
	Model 2	1.62 (1.48–1.77)***	1.66 (1.52–1.82)***	1.70 (1.55–1.86)***	1.63 (1.49–1.78)***	1.65 (1.52–1.80)***	1.61 (1.47–1.75)***	1.61 (1.48–1.74)***
Hypertriglyceridemia	Crude	1.62 (1.48–1.77)***	1.85 (1.68–2.03)***	1.96 (1.78–2.15)***	1.72 (1.57–1.88)***	1.89 (1.72–2.07)***	1.53 (1.40–1.67)***	1.66 (1.53–1.82)***
	Model 1	1.66 (1.51–1.83)***	1.80 (1.63–1.99)***	1.94 (1.74–2.16)***	1.76 (1.60–1.93)***	1.91 (1.73–2.11)***	1.62 (1.48–1.78)***	1.71 (1.56–1.88)***
	Model 2	1.66 (1.51–1.83)***	1.81 (1.63–2.00)***	1.96 (1.76–2.18)***	1.76 (1.60–1.94)***	1.92 (1.74–2.12)***	1.62 (1.48–1.78)***	1.72 (1.57–1.89)***

*WC* waist circumference (cm), *WHtR* waist-to-height ratio, *TFM* trunk fat mass (kg), *%TFM* percent trunk fat mass (%), *WBTFM* whole-body total fat mass (kg), *%WBTFM* percent whole-body total fat mass (%). Model 1: adjusted for age, drinking and smoking; Model 2: adjusted for age, drinking, smoking, exercise, income, town, education, occupation and stress. Complex-samples multiple logistic regression analyses with adjustments were performed using weight, cluster and stratification parameters to consider the complex-sample survey data. Values are presented as odds ratios with 95% confidence intervals. *p < 0.05; **p < 0.01; ***p < 0.001.

**Table 3:** Association of CVD with anthropometric and body composition indices in women.

CVD	Analysis	BMI	WC	WHtR	TFM	%TFM	WBTFM	%WBTFM
Hypertension	Crude	1.79 (1.64–1.95)***	2.31 (2.12–2.52)***	2.95 (2.7–3.21)***	1.83 (1.68–1.99)***	2.29 (2.06–2.54)***	1.42 (1.31–1.54)***	1.69 (1.54–1.85)***
	Model 1	1.82 (1.66–1.98)***	1.82 (1.67–1.99)***	1.90 (1.73–2.09)***	1.84 (1.69–2.01)***	1.78 (1.60–1.99)***	1.67 (1.53–1.82)***	1.54 (1.39–1.70)***
	Model 2	1.80 (1.64–1.98)***	1.79 (1.64–1.97)***	1.88 (1.70–2.08)***	1.83 (1.67–2.00)***	1.76 (1.57–1.96)***	1.66 (1.52–1.81)***	1.52 (1.37–1.68)***
Hyperlipidemia	Crude	1.62 (1.49–1.76)***	1.86 (1.71–2.03)***	2.08 (1.91–2.27)***	1.71 (1.56–1.87)***	2.06 (1.84–2.31)***	1.40 (1.29–1.52)***	1.56 (1.41–1.73)***
	Model 1	1.53 (1.39–1.69)***	1.52 (1.37–1.69)***	1.52 (1.36–1.71)***	1.62 (1.47–1.79)***	1.65 (1.47–1.85)***	1.46 (1.32–1.60)***	1.37 (1.24–1.52)***
	Model 2	1.53 (1.39–1.69)***	1.54 (1.39–1.72)***	1.58 (1.40–1.78)***	1.61 (1.46–1.77)***	1.62 (1.44–1.83)***	1.43 (1.30–1.57)***	1.35 (1.21–1.49)***
Diabetes	Crude	1.65 (1.51–1.81)***	2.18 (1.96–2.43)***	2.55 (2.28–2.84)***	1.69 (1.53–1.87)***	1.84 (1.62–2.09)***	1.30 (1.18–1.43)***	1.32 (1.18–1.48)***
	Model 1	1.58 (1.43–1.75)***	1.83 (1.63–2.07)***	1.89 (1.67–2.14)***	1.62 (1.46–1.81)***	1.41 (1.24–1.60)***	1.35 (1.22–1.49)***	1.13 (1.02–1.26)*
	Model 2	1.54 (1.39–1.71)***	1.81 (1.60–2.05)***	1.86 (1.63–2.12)***	1.60 (1.44–1.79)***	1.38 (1.22–1.57)***	1.32 (1.20–1.46)***	1.11 (1.00–1.24)*
Hypercholesterolemia	Crude	1.62 (1.50–1.75)***	1.86 (1.72–2.02)***	2.07 (1.90–2.24)***	1.70 (1.56–1.85)***	2.00 (1.80–2.23)***	1.44 (1.33–1.55)***	1.61 (1.47–1.78)***
	Model 1	1.53 (1.41–1.66)***	1.55 (1.42–1.70)***	1.59 (1.44–1.76)***	1.60 (1.46–1.75)***	1.66 (1.49–1.85)***	1.48 (1.36–1.61)***	1.46 (1.32–1.60)***
	Model 2	1.52 (1.40–1.65)***	1.55 (1.42–1.70)***	1.61 (1.46–1.78)***	1.58 (1.45–1.73)***	1.64 (1.47–1.83)***	1.46 (1.34–1.59)***	1.43 (1.30–1.58)***
Hypo-HDL cholesterolemia	Crude	1.51 (1.39–1.65)***	1.70 (1.56–1.84)***	1.76 (1.62–1.91)***	1.52 (1.40–1.66)***	1.58 (1.45–1.71)***	1.35 (1.24–1.45)***	1.32 (1.23–1.42)***
	Model 1	1.45 (1.33–1.58)***	1.57 (1.44–1.72)***	1.62 (1.47–1.79)***	1.46 (1.34–1.59)***	1.43 (1.30–1.56)***	1.35 (1.25–1.46)***	1.24 (1.15–1.34)***
	Model 2	1.44 (1.32–1.57)***	1.55 (1.42–1.70)***	1.59 (1.43–1.75)***	1.46 (1.34–1.59)***	1.41 (1.29–1.54)***	1.35 (1.25–1.45)***	1.22 (1.13–1.32)***
Hypertriglyceridemia	Crude	1.65 (1.51–1.79)***	1.91 (1.75–2.09)***	2.09 (1.92–2.28)***	1.74 (1.60–1.89)***	2.02 (1.84–2.22)***	1.43 (1.31–1.56)***	1.54 (1.40–1.69)***
	Model 1	1.57 (1.43–1.73)***	1.68 (1.52–1.86)***	1.78 (1.59–1.98)***	1.65 (1.51–1.81)***	1.74 (1.56–1.94)***	1.46 (1.33–1.60)***	1.41 (1.27–1.57)***
	Model 2	1.55 (1.41–1.71)***	1.64 (1.48–1.82)***	1.73 (1.55–1.94)***	1.64 (1.50–1.80)***	1.72 (1.54–1.92)***	1.45 (1.32–1.59)***	1.39 (1.25–1.55)***

*WC* waist circumference (cm), *WHtR* waist-to-height ratio, *TFM* trunk fat mass (kg), *%TFM* percent trunk fat mass (%), *WBTFM* whole-body total fat mass (kg), *%WBTFM* percent whole-body total fat mass (%). Model 1: adjusted for age, drinking and smoking; Model 2: adjusted for age, drinking, smoking, exercise, income, town, education, occupation and stress. Complex-samples multiple logistic regression analyses with adjustments were performed using weight, cluster and stratification parameters to consider the complex-sample survey data. Values are presented as odds ratios with 95% confidence intervals. *p < 0.05; **p < 0.01; ***p < 0.001.

In women, hypertension tended to be more strongly associated with WHtR in all models. Specifically, WHtR was slightly more strongly associated with hypertension than TFM in adjusted model 1 (OR = 1.90 [1.73–2.09] and OR = 1.84 [1.69–2.01]) and in adjusted model 2 (OR = 1.88 [1.70–2.08] and OR = 1.83 [1.67–2.00]). Hyperlipidemia was more closely associated with WHtR (OR = 2.08 [1.91–2.27]) and %TFM (OR = 2.06 [1.84–2.31]) than the other indices in the crude analysis and was more related to TFM (OR = 1.62 [1.47–1.79] and %TFM (OR = 1.65 [1.47–1.85]) in adjusted model 1, but hyperlipidemia was similarly associated with %TFM, TFM, and WHtR in adjusted model 2 (OR = 1.62 [1.44–1.83], OR = 1.61 [1.46–1.77], and OR = 1.58 [1.40–1.78, respectively). Additionally, diabetes was more strongly associated with WHtR in all models, and the index was slightly more strongly associated with diabetes than WC in adjusted model 1 (OR = 1.89 [1.67–2.14] and OR = 1.83 [1.63–2.07]) and adjusted model 2 (OR = 1.86 [1.63–2.12] and OR = 1.81 [1.60–2.05]). Hypercholesterolemia was more strongly related to WHtR and %TFM than other indices on crude analysis (OR = 2.07 [1.90–2.24] and OR = 2.00 [1.80–2.23]) and in adjusted model 2 (OR = 1.61 [1.46–1.78] and OR = 1.64 [1.47–1.83]). However, %TFM was slightly more associated with the disease than WHtR in model 1 (OR = 1.66 [1.49–1.85] and OR = 1.59 [1.44–1.76]). Hypo-HDL cholesterolemia was more strongly associated with WHtR and WC in all models, and the association of WHtR was slightly stronger than that of WC. Hypertriglyceridemia was more strongly associated with WHtR and %TFM in all models, and WHtR was slightly more strongly associated with hypertriglyceridemia than %TFM in all models. Overall, in women, the metabolic risk factors tended to be more closely associated with WHtR than the other indices, except for TFM and %TFM in adjusted models 1 and 2 in hyperlipidemia and %TFM in adjusted models 1 and 2 in hypercholesterolemia. Comparing all indices for anthropometry and body composition, WHtR was more strongly associated with hypertension, diabetes, hypo-HDL cholesterolemia, and hypertriglyceridemia.

## Discussion

Although numerous studies have been performed to examine the association of obesity and adiposity with CVD and metabolic risk factors in public health and epidemiology, few studies have compared anthropometric and body composition indices to identify CVD and metabolic risk factors^12–17^. For example, Bosy-Westphal et al.^12^ compared the value of percent body fat mass, BMI, WC, and WHtR in predicting metabolic risk factors based on data from 355 adults from the Kiel Obesity Prevention Study. They argued that body fat mass has no benefits in identifying metabolic risk factors compared with BMI and WC because the magnitude of the association of WHtR and WC with risk factors was slightly higher than or equal to that of percent body fat mass and BMI. They also found that WHtR showed the best value on receiver operating characteristic curve analysis in identifying the prevalence of 2 ≥ component traits among the triglyceride, blood pressure, and glucose levels. Weber et al.^13^ reported that body fat, the mass index and the lean body mass index had no advantages over BMI in revealing metabolic syndrome in children and adolescents from the US, BMI was a suitable tool for screening for cardiometabolic risks, and the use of body composition indices determined by DXA was not cost-effective in the clinical setting. Zhang et al.^14^ analyzed BMI, WC, hip circumference (HC), and WHtR based on anthropometry and body fat, percent body fat, trunk fat, and percent trunk fat based on BIA to identify metabolic risk factors and metabolic syndrome in Chinese adults (2780 women and 1160 men). They reported that the strongest indicator of these diseases was WHtR, and the value of indices based on BIA was much lower than that of indices based on anthropometry. Additionally, they documented that WHtR had the strongest association with hypertension, dyslipidemia, hyperuricemia, diabetes, and metabolic syndrome in men and with dyslipidemia, diabetes, and metabolic syndrome in women. Sun et al.^15^ examined the correlation between anthropometric and body composition indices to predict obesity-related metabolic risk factors in 8,773 adults from the US and reported that the correlations of risk factors with fat mass or percent fat mass measured in the trunk and whole body were similar or equal to those of WC and BMI and that the use of anthropometric indices was thus comparable to that of body composition indices determined by DXA. A cross-sectional study by Lindsay et al.^16^ found that BMI could be used as a reasonable indicator for body fat mass or percent fat mass determined by DXA in Pima Indian children. Another study by Vatanparast et al.^17^ suggested that the abdominal fat mass index was the best predictor of blood lipid levels in 423 white postmenopausal females in Canada, but the index was similar or equal to WC in predicting the blood lipid profile. Therefore, they argued that WC was an ideal indicator of blood lipid levels in terms of a cost-effective means of CVD screening in primary healthcare. The main findings of this study are that WHtR from anthropometric indices tends to be equally or more strongly associated with hyperlipidemia, diabetes, hypo-HDL cholesterolemia, and hypertriglyceridemia in men and hypertension, diabetes, hypo-HDL cholesterolemia, and hypertriglyceridemia in women compared to other indices. This observation is in line with previous studies^12–15^. We have now confirmed the results in a large-scale Korean population.

Many studies have recommended that BMI can be used as a representative index in studies on obesity and related diseases. However, BMI is not suitable as an indicator of health outcomes or many diseases because BMI is not considered to be related to the detrimental influence of intra-abdominal fat on mortality and morbidity^1^. BMI cutoff points are not appropriate for use worldwide because most associations between BMI and TFM or waist fat mass were not determined in homogenous populations, and these associations differ according to age and ethnic group^11,16,18–20^. There is a need to develop a more reliable index and algorithm for the quantification or identification of CVD, hypertension, and diabetes on a global scale^21^. For these reasons, several anthropometric indices, such as WC, HC, WHR, and WHtR, have been substituted for BMI as indicators of chronic diseases in the screening step and show strong associations with various chronic diseases^7,13,22–25^. However, one of the most important issues of studies on associations between obesity-related chronic diseases and anthropometric or body composition indices is that the best predictor of the diseases remains unclear. Many studies have suggested that the strongest predictor among anthropometric and body composition indices differs according to metabolic risk factors, age, sex, ethnicity, and country, among others^6–9,26^. Furthermore, studies based on the same ethnic group or country may differ according to the data or population studied, sex, and age group. Specifically, for diabetes, the strongest indicator was WHR in Taiwanese adults aged 45–64 y^27^, in Australian adults^28^, in Iraqi adult men and women^29^, in Iranian adult men^30^, and in Korean men^31,32^. However, WC was the best predictor in sub-Saharan Africa^33^ and in Singapore residents of Chinese, Malay or Indian ethnicity^34^. WHtR was the most strongly associated with diabetes in women from the US^35^, in Chinese adult men and women^36,37^, in Korean adult men and women^38^, and in meta-analyses of several ethnic groups and countries^39–41^. The strongest indicators were the waist-to-thigh ratio (WTR) in adult men from the US^42^ and predicted fat mass in men from the US^35^. WC and WHtR were the best predictors in Germany^43^ and in Korean adult men and women^44^. Other studies have suggested that the strongest predictors of diabetes are abdominal fat mass in Korean adult men and women^45^ and rib-to-hip circumference in Korean women^31,32^. In hypertension, WHR was the strongest predictor in Australian adults^28^ and in Iranian adult men^30^, while WC was the best predictor in Canadian adults^46^ and in Italian adults^47^. WHtR was the best indicator in Iraqi adult men and women^39^, in Korean adults^38^ and Korean women^48^, and in meta-analyses of several ethnic groups and countries^38,49^. Furthermore, the strongest indicators of hypertension were BMI in Singapore residents of Chinese, Malay, or Indian ethnicity^34^, BMI and WHtR in Chinese adults^50^, a body shape index (ABSI) in Portuguese adolescents^51^, and rib circumference in Korean adults^48,52^. In the present study, our findings indicate that WHtR is more strongly associated with most metabolic risk factors than other indices. This is in accordance with previous studies of diabetes^36–41^ and hypertension^38–40,49^.

The results of this study have several limitations. In this study, cause-effect relationships cannot be described due to the cross-sectional study design. Furthermore, we cannot support additional statistical analyses, such as determining the area under the receiver operating characteristic (ROC) curve or performing the Wald test, the so-called Z-test for determining differences between two beta coefficients from independent models, because the data are complex-sample survey data. However, this study has strengths. The findings and statistical results in the present study are powerful due to the large scale of the study. A nationally representative sample of the Korean population supported by the KNHANES was used in this study, and this sample was collected from all provinces in South Korea over a long period of time. To our knowledge, this is the first report of a comparison between anthropometric and body composition indices based on a large-scale study in Korea.

In conclusion, we suggest that the use of body fat mass indices is not suitable for identifying metabolic abnormalities on the large-scale screening of the Korean adult population because anthropometric indices may be equal to or better than body composition indices in terms of the power for identifying metabolic risk factors. Additionally, WHtR was similar to or more associated with hyperlipidemia, diabetes, hypo-HDL cholesterolemia, and hypertriglyceridemia in men and hypertension, diabetes, hypo-HDL cholesterolemia, and hypertriglyceridemia in women compared to other indices in the Korean population.

## Methods

### Subjects and data source

This study was based on data from the Korea National Health and Nutrition Examination Survey (KNHANES), which is a nationwide, cross-sectional survey that has been conducted by the Korea Centers for Disease Control and Prevention (KCDC) since 1998 to evaluate the health and nutritional status of adults and children in Korea. Survey subjects were selected using a multistage, stratified and clustered random sampling method to reflect the characteristics of the overall Korean population. The detailed descriptions and microdata of the KNHANES are offered on the website (http://knhanes.cdc.go.kr/)^53^.

**Figure 1:**
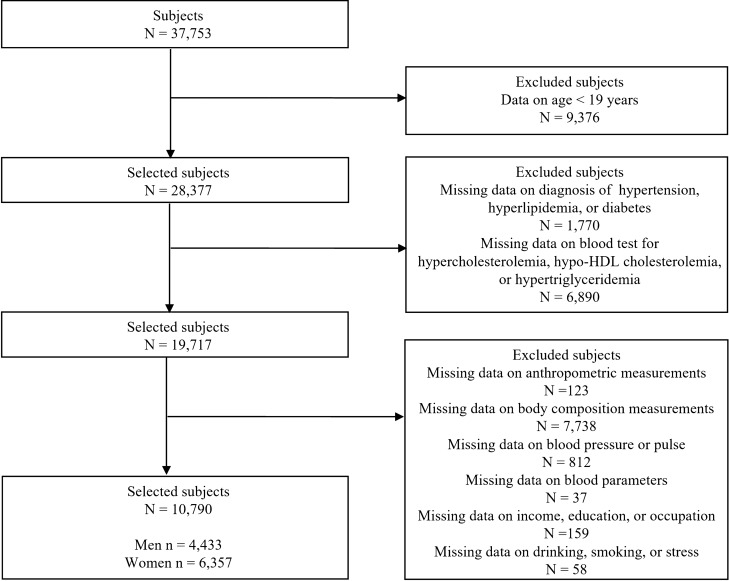
Sample selection procedure used in this study.

The KNHANES collected body composition measurements from 2008 to 2011. Therefore, we used the KNHANES data from 2008 to 2011 (KNHANES IV–V, 2008–2011). The KNHANES IV–V 2008–2011 includes 37,753 (men = 17,195, women = 20,558) subjects. We selected subjects based on inclusion and exclusion criteria. We included subjects aged between 19 and 80 years. Finally, we selected a total of 10,790 subjects, which included 4433 men and 6357 women. Figure 1 shows the sample selection procedure according to the flow of inclusion and exclusion and the number of subjects in detail. Table 1 shows the demographic characteristics of the subjects used in this study.

All survey subjects were required to sign an informed consent forms, and all subjects signed informed consent forms. The KNHANES IV–V 2008–2011 was approved by the Institutional Review Board of the KCDC (2008-04EXP-01-C, 2009-01CON-03-2C, 2010-02CON-21-C, 2011-02CON-06-C) and conducted in accordance with the Declaration of Helsinki. This study obtained ethics approval from the Institutional Review Board of the Korea Institute of Oriental Medicine for analysis of the open-source database of the KNHANES IV–V (IRB No. I-1909/007–003). All methods used in this study were carried out in accordance with relevant guidelines and regulations.

### Definitions

Information regarding subjects with diagnoses made by physicians, such as hypertension, hyperlipidemia, and diabetes, was collected from health interviews. Subjects with hypertension were defined as those who answered “Yes” to the question “Do you have hypertension diagnosed by a physician?” via face-to-face interviews with well-trained staff members, and subjects without hypertension were defined as those who answered “No” or “Not applicable”, according to the guidelines of the KCDC^53^. Subjects with hyperlipidemia and diabetes were defined in the same manner as subjects with hypertension. Hypercholesterolemia, hypo-high-density lipoprotein (HDL) cholesterolemia, and hypertriglyceridemia were determined based on laboratory blood tests performed during health examinations. We defined hypercholesterolemia as a total cholesterol ≥ 240 mg/dl or the current use of any cholesterol medication. Hypo-HDL cholesterolemia was defined as HDL cholesterol < 40 mg/dl, and hypertriglyceridemia was defined as triglyceride ≥ 200 mg/dl. All demographic characteristics in the normal and patient groups are described in the Supplementary Materials.

### Anthropometric, body composition, and laboratory blood test data

In the KNHANES, weight and height were measured according to standard protocols to the nearest 0.1 cm (Seca 225, Seca, Germany) and 0.1 kg (GL-6000–20, G-tech, Korea), respectively. BMI was defined as weight (kg) divided by height squared (m^2^). WC was measured to the nearest 0.1 cm using a measuring tape at the midpoint between the iliac crest and the lowest rib. Blood pressure was measured in the right arm and defined by the mean of the second and third values obtained using a standard mercury sphygmomanometer (Baumanometer; WA Baum Co., Copiague, NY, USA).

Blood sampling was conducted after fasting for at least 8 h. The serum levels of total cholesterol, triglyceride, HDL-C, glucose, aspartate aminotransferase (AST), alanine aminotransferase (ALT), and creatinine were measured using an automatic analyzer, such as an Advia 1650/2400 (Siemens, New York, NY, USA) or Hitachi Automatic Analyzer 7600 (Hitachi, Tokyo, Japan).

Body composition indices were measured with a fan-beam densitometer (DISCOVERY-W, fan-beam densitometer, Hologic, Inc., USA) using DXA according to the procedures provided by the manufacturer. Before the examination, all subjects removed jewelry and metal that they were wearing to avoid interfering with the DXA examination.

For smoking status, alcohol consumption, and physical activity categorization, smoking was classified into three levels, which were “smoking” if the subjects were smoking currently, “quit smoking” if they quit smoking, and “never smoked” if they had never smoked; drinking was classified into six levels according to the drinking frequency over the last year, which were “not at all for the past one year”, “less than once a month”, “once a month”, “2 to 4 times a month”, “2 or 3 times a week” and, “4 or more times a week”; physical activity was also classified into eight levels according to the exercise frequency for one week, which were “not at all”, “once a week”, “2 times a week”, “3 times a week”, “4 times a week”, “5 times a week”, “6 times a week”, and “every day”.

### Statistical analysis

All statistical analyses were implemented using complex-sample procedures in SPSS Statistics 23 for Windows (SPSS, Inc., Chicago, IL, US) to take into account the complex-sample survey data. Weight, cluster and stratification variables for complex-sample analysis were provided by the KNHANES. A significance level was determined at α = 0.05 for all statistical tests. Continuous variables are represented as the mean ± standard error (SE), and categorical variables are represented as the percentage (SE). Complex-samples general linear models were adopted for continuous variables, and Rao-Scott chi-square tests were adopted for categorical variables to compare differences between the normal groups and each of the six metabolic risk factor groups. Complex-samples general linear models and Rao-Scott chi-square tests were used for continuous and categorical variables, respectively, to compare differences between men and women. Regarding the six metabolic risk factors, complex-sample binary logistic linear models were applied to evaluate the association of each risk factor with body measurements after standardization of the data by sex. Complex-samples multiple logistic regression models were established to assess the relationship between each risk factor and body measurements, with multiple covariates accounting for several various confounders by sex. Three models were developed as follows: model 1 was crude; model 2 included adjustments for age, drinking and smoking; and model 3 included adjustments for age, drinking, smoking, exercise, income, town, education, occupation and stress. Odds ratios are presented with 95% confidence intervals and p-values for each model by sex.

## Supplementary Information

Supplementary Tables

## Data Availability

This study was based on data from the Korea National Health and Nutrition Examination Survey (KNHANES), which is a nationwide, cross-sectional survey that has been conducted by the Korea Centers for Disease Control and Prevention (KCDC) since 1998 to evaluate the health and nutritional status of adults and children in Korea. The detailed descriptions and microdata of the KNHANES are offered on the website (http://knhanes.cdc.go.kr/). Data are available from the KNHANES by the Korea Centers for Disease Control and Prevention (http://knhanes.cdc.go.kr/ and https://knhanes.cdc.go.kr/knhanes/main.do).
