# Supplementary material for: Comparison of anthropometric and body composition indices in the identification of metabolic risk factors
Source: Sci Rep. 2021 May 11;11:9931. doi: 10.1038/s41598-021-89422-x (PMC8113511; doi:10.1038/s41598-021-89422-x)
Supplement: Supplementary file 1 — Supplementary Tables [file 41598_2021_89422_MOESM1_ESM.docx]

**Supplementary materials**

**Title: Comparison of anthropometric and body composition indices in the identification of metabolic risk factors**

Authors: **Bum Ju Lee**^1*^ and Mi Hong Yim^1^

^1^ Future Medicine Division, Korea Institute of Oriental Medicine, Republic of Korea

^*^ Corresponding author: Bum Ju Lee (E-mail: bjlee@kiom.re.kr)

**Supplementary Table 1.** Basic characteristics of hypertension and normotension groups.

| Variables | Men |  |  |  | Women |  |  |  |
| --- | --- | --- | --- | --- | --- | --- | --- | --- |
|  | P-value† |  | Normotension | Hypertension | P-value† |  | Normotension | Hypertension |
| Number of subjects |  |  | 3,472 | 961 |  |  | 5,036 | 1,321 |
| Age (years) | <.001 |  | 41.19 ± 0.33 | 57.26 ± 0.56 | <.001 |  | 41.90 ± 0.31 | 63.23 ± 0.39 |
| BMI (kg/m^2^) | <.001 |  | 23.79 ± 0.06 | 25.24 ± 0.16 | <.001 |  | 22.93 ± 0.07 | 25.22 ± 0.12 |
| Waist circumference (cm) | <.001 |  | 82.95 ± 0.20 | 88.56 ± 0.42 | <.001 |  | 76.55 ± 0.20 | 84.96 ± 0.35 |
| Waist-to-height ratio | <.001 |  | 0.48 ± 0.001 | 0.53 ± 0.002 | <.001 |  | 0.48 ± 0.001 | 0.56 ± 0.002 |
| SBP (mmHg) | <.001 |  | 119.29 ± 0.33 | 133.05 ± 0.66 | <.001 |  | 112.11 ± 0.33 | 134.72 ± 0.56 |
| DBP (mmHg) | <.001 |  | 79.99 ± 0.26 | 84.81 ± 0.50 | <.001 |  | 73.18 ± 0.22 | 80.82 ± 0.35 |
| Pulse rate (beats per 15 seconds) | .300 |  | 17.55 ± 0.05 | 17.67 ± 0.11 | .011 |  | 17.95 ± 0.06 | 17.75 ± 0.07 |
| Hemoglobin (mg/dl) | <.001 |  | 15.39 ± 0.02 | 14.97 ± 0.05 | <.001 |  | 12.94 ± 0.02 | 13.10 ± 0.04 |
| Cholesterol (mg/dl) | .161 |  | 187.64 ± 0.77 | 185.55 ± 1.26 | <.001 |  | 185.36 ± 0.63 | 195.79 ± 1.38 |
| Triglyceride (mg/dl) | <.001 |  | 151.38 ± 2.73 | 178.31 ± 5.30 | <.001 |  | 103.19 ± 1.32 | 146.30 ± 2.81 |
| Glucose (mg/dl) | <.001 |  | 95.74 ± 0.46 | 108.53 ± 1.25 | <.001 |  | 92.24 ± 0.28 | 105.13 ± 1.01 |
| AST (IU/L) | .001 |  | 24.50 ± 0.29 | 27.12 ± 0.71 | <.001 |  | 19.23 ± 0.13 | 22.98 ± 0.32 |
| ALT (IU/L) | .383 |  | 27.13 ± 0.41 | 27.91 ± 0.78 | <.001 |  | 16.60 ± 0.22 | 20.58 ± 0.40 |
| Creatinine (mg/dl) | .002 |  | 0.95 ± 0.003 | 1.02 ± 0.02 | <.001 |  | 0.70 ± 0.002 | 0.75 ± 0.01 |
| Trunk fat mass (kg) | <.001 |  | 8.29 ± 0.08 | 10.10 ± 0.16 | <.001 |  | 9.30 ± 0.07 | 11.69 ± 0.14 |
| Percent trunk fat mass (%) | <.001 |  | 23.82 ± 0.18 | 27.60 ± 0.27 | <.001 |  | 32.32 ± 0.17 | 37.60 ± 0.25 |
| Whole-body total fat mass (kg) | <.001 |  | 15.40 ± 0.14 | 17.50 ± 0.28 | <.001 |  | 18.91 ± 0.12 | 21.11 ± 0.22 |
| Percent whole-body total fat mass (%) | <.001 |  | 21.76 ± 0.15 | 24.34 ± 0.23 | <.001 |  | 32.73 ± 0.14 | 35.47 ± 0.21 |
| Mean sleep duration (hours) | .033 |  | 6.91 ± 0.03 | 6.78 ± 0.05 | <.001 |  | 171.26 ± 0.14 | 168.12 ± 0.29 |
| Region (city) | .315 |  |  |  | .002 |  |  |  |
| Seoul |  | 21.58 (1.10) | 21.4 (1.16) | 22.54 (1.77) |  | 20.8 (0.91) | 21.39 (0.98) | 17.87 (1.52) |
| Busan |  | 7.89 (0.74) | 7.91 (0.75) | 7.82 (1.53) |  | 7.82 (0.84) | 7.82 (0.78) | 7.82 (1.84) |
| Daegu |  | 5.15 (0.71) | 5.24 (0.76) | 4.69 (0.90) |  | 5.11 (0.70) | 5.34 (0.75) | 3.99 (0.74) |
| Incheon |  | 5.30 (0.58) | 5.22 (0.64) | 5.73 (0.93) |  | 5.15 (0.52) | 5.17 (0.55) | 5.02 (0.81) |
| Gwangju |  | 2.27 (0.69) | 2.47 (0.78) | 1.20 (0.48) |  | 2.21 (0.60) | 2.37 (0.66) | 1.41 (0.45) |
| Daejeon |  | 3.18 (0.68) | 3.28 (0.69) | 2.70 (0.96) |  | 3.36 (0.68) | 3.32 (0.67) | 3.58 (1.03) |
| Ulsan |  | 2.11 (0.64) | 2.28 (0.72) | 1.18 (0.50) |  | 2.55 (0.75) | 2.65 (0.81) | 2.02 (0.76) |
| Gyeonggi-do |  | 22.85 (1.11) | 23.02 (1.20) | 21.95 (2.04) |  | 22.98 (0.97) | 23.54 (1.05) | 20.20 (1.67) |
| Gangwon-do |  | 2.34 (0.39) | 2.12 (0.37) | 3.47 (0.80) |  | 2.32 (0.50) | 2.20 (0.53) | 2.94 (0.70) |
| Chungcheongbuk-do |  | 3.36 (0.61) | 3.28 (0.66) | 3.76 (0.76) |  | 3.34 (0.57) | 3.16 (0.61) | 4.24 (0.61) |
| Chungcheongnam-do |  | 3.95 (0.66) | 3.73 (0.69) | 5.10 (0.99) |  | 4.59 (0.67) | 4.24 (0.72) | 6.29 (1.02) |
| Jeollabuk-do |  | 2.98 (0.47) | 2.91 (0.52) | 3.35 (0.62) |  | 3.06 (0.47) | 2.56 (0.45) | 5.50 (0.92) |
| Jeollanam-do |  | 2.80 (0.48) | 2.79 (0.48) | 2.89 (0.77) |  | 3.24 (0.51) | 3.10 (0.52) | 3.93 (0.81) |
| Gyeongsangbuk-do |  | 5.58 (0.72) | 5.55 (0.82) | 5.78 (0.75) |  | 5.79 (0.74) | 5.55 (0.83) | 6.99 (1.37) |
| Gyeongsangnam-do |  | 6.83 (0.95) | 7.10 (1.03) | 5.39 (1.12) |  | 5.73 (0.77) | 5.55 (0.80) | 6.62 (1.13) |
| Jeju-do |  | 1.82 (0.67) | 1.70 (0.61) | 2.44 (1.23) |  | 1.95 (0.96) | 2.02 (0.96) | 1.58 (0.97) |
| Town | .007 |  |  |  | <.001 |  |  |  |
| Dong (city) |  | 80.62 (1.83) | 81.42 (1.83) | 76.35 (2.56) |  | 80.37 (1.81) | 82.40 (1.73) | 70.27 (2.77) |
| Eup, Myeon (rural) |  | 19.38 (1.83) | 18.58 (1.83) | 23.65 (2.56) |  | 19.63 (1.81) | 17.60 (1.73) | 29.73 (2.77) |
| Income | .642 |  |  |  | .870 |  |  |  |
| 1st quartile (low) |  | 25.78 (0.96) | 25.80 (1.06) | 25.64 (1.79) |  | 26.42 (0.85) | 26.45 (0.94) | 26.29 (1.59) |
| 2nd quartile (lower-middle) |  | 26.27 (0.89) | 26.58 (0.96) | 24.63 (1.87) |  | 25.37 (0.74) | 25.56 (0.83) | 24.44 (1.42) |
| 3rd quartile (upper-middle) |  | 23.33 (0.76) | 22.99 (0.83) | 25.13 (1.80) |  | 24.97 (0.73) | 24.95 (0.84) | 25.06 (1.46) |
| 4th quartile (high) |  | 24.62 (0.98) | 24.62 (1.05) | 24.59 (1.78) |  | 23.24 (0.84) | 23.05 (0.91) | 24.21 (1.56) |
| Education | <.001 |  |  |  | <.001 |  |  |  |
| Elementary school or less |  | 11.98 (0.60) | 9.63 (0.59) | 24.53 (1.68) |  | 25.50 (0.89) | 17.28 (0.78) | 66.39 (1.68) |
| Middle school |  | 10.32 (0.52) | 8.93 (0.54) | 17.71 (1.52) |  | 9.50 (0.44) | 9.10 (0.49) | 11.47 (1.09) |
| High school |  | 40.35 (1.06) | 41.82 (1.15) | 32.50 (2.03) |  | 37.45 (0.96) | 41.39 (1.07) | 17.79 (1.31) |
| University or higher |  | 37.36 (1.13) | 39.63 (1.22) | 25.25 (1.93) |  | 27.56 (0.89) | 32.22 (0.97) | 4.35 (0.75) |
| Occupation | <.001 |  |  |  | <.001 |  |  |  |
| Managers, professionals and related workers |  | 17.91 (0.79) | 18.73 (0.89) | 13.49 (1.56) |  | 10.23 (0.51) | 11.94 (0.60) | 1.75 (0.50) |
| Clerks |  | 11.60 (0.59) | 12.07 (0.66) | 9.08 (1.19) |  | 6.39 (0.36) | 7.37 (0.41) | 1.51 (0.49) |
| Service workers and sale workers |  | 12.84 (0.66) | 13.41 (0.73) | 9.78 (1.26) |  | 14.55 (0.62) | 15.38 (0.68) | 10.43 (1.14) |
| Skilled agricultural, forestry and fishery workers |  | 7.71 (0.89) | 7.06 (0.88) | 11.16 (1.49) |  | 5.51 (0.66) | 4.54 (0.55) | 10.33 (1.62) |
| Craft, plant, machine operators and assemblers |  | 20.09 (0.82) | 20.79 (0.94) | 16.38 (1.60) |  | 2.30 (0.23) | 2.32 (0.25) | 2.21 (0.50) |
| Elementary occupations |  | 7.25 (0.49) | 7.02 (0.54) | 8.48 (1.08) |  | 8.40 (0.44) | 7.83 (0.49) | 11.24 (1.09) |
| Unemployed |  | 22.61 (0.91) | 20.92 (0.99) | 31.63 (1.79) |  | 52.62 (0.86) | 50.63 (0.94) | 62.54 (1.95) |
| Drinking | <.001 |  |  |  | <.001 |  |  |  |
| Not at all for the past one year |  | 13.01 (0.65) | 12.04 (0.69) | 18.19 (1.57) |  | 33.08 (0.84) | 28.96 (0.91) | 53.6 (1.75) |
| Less than once a month |  | 9.68 (0.61) | 10.09 (0.68) | 7.48 (1.16) |  | 26.72 (0.68) | 27.82 (0.74) | 21.21 (1.43) |
| Once a month |  | 9.23 (0.55) | 9.35 (0.60) | 8.58 (1.09) |  | 11.73 (0.49) | 12.53 (0.57) | 7.75 (0.87) |
| 2 to 4 times a month |  | 30.70 (0.87) | 32.27 (1.00) | 22.35 (1.82) |  | 19.48 (0.66) | 21.11 (0.75) | 11.37 (1.10) |
| 2 or 3 times a week |  | 24.23 (0.78) | 24.57 (0.91) | 22.46 (1.66) |  | 6.94 (0.44) | 7.58 (0.51) | 3.76 (0.67) |
| 4 or more times a week |  | 13.14 (0.62) | 11.68 (0.65) | 20.94 (1.72) |  | 2.05 (0.22) | 1.99 (0.24) | 2.3 (0.52) |
| Smoking | <.001 |  |  |  | .001 |  |  |  |
| Smoking |  | 46.4 (0.99) | 49.24 (1.07) | 31.27 (2.02) |  | 6.58 (0.42) | 6.92 (0.48) | 4.89 (0.76) |
| Quit smoking |  | 33.4 (0.87) | 29.62 (0.93) | 53.53 (1.90) |  | 6.39 (0.41) | 6.85 (0.48) | 4.12 (0.65) |
| Never smoked |  | 20.2 (0.76) | 21.14 (0.87) | 15.20 (1.37) |  | 87.03 (0.58) | 86.23 (0.67) | 90.99 (0.98) |
| Stress | <.001 |  |  |  | <.001 |  |  |  |
| Extremely |  | 4.10 (0.34) | 4.11 (0.38) | 4.06 (0.79) |  | 5.26 (0.34) | 5.14 (0.36) | 5.91 (0.77) |
| Very |  | 22.36 (0.79) | 22.74 (0.89) | 20.36 (1.66) |  | 26.32 (0.72) | 26.90 (0.81) | 23.41 (1.40) |
| Slightly |  | 59.50 (0.88) | 60.54 (0.97) | 53.98 (1.88) |  | 56.18 (0.75) | 57.32 (0.83) | 50.48 (1.71) |
| Rarely |  | 14.04 (0.59) | 12.62 (0.63) | 21.61 (1.56) |  | 12.24 (0.48) | 10.64 (0.53) | 20.2 (1.22) |
| Exercise | <.001 |  |  |  | <.001 |  |  |  |
| Not at all |  | 55.8 (0.97) | 54.12 (1.09) | 64.77 (1.84) |  | 74.19 (0.80) | 72.65 (0.88) | 81.84 (1.34) |
| Once a week |  | 15.66 (0.75) | 16.82 (0.86) | 9.45 (1.19) |  | 6.56 (0.39) | 7.17 (0.45) | 3.51 (0.64) |
| 2 times a week |  | 9.72 (0.54) | 10.36 (0.61) | 6.34 (0.92) |  | 5.24 (0.34) | 5.47 (0.38) | 4.06 (0.66) |
| 3 times a week |  | 7.26 (0.45) | 7.36 (0.51) | 6.72 (1.05) |  | 5.74 (0.41) | 5.98 (0.46) | 4.58 (0.72) |
| 4 times a week |  | 3.19 (0.33) | 3.16 (0.36) | 3.31 (0.78) |  | 2.23 (0.26) | 2.33 (0.29) | 1.71 (0.50) |
| 5 times a week |  | 3.49 (0.34) | 3.49 (0.39) | 3.51 (0.74) |  | 2.78 (0.25) | 3.06 (0.28) | 1.41 (0.38) |
| 6 times a week |  | 2.12 (0.27) | 2.13 (0.30) | 2.03 (0.62) |  | 0.97 (0.14) | 1.00 (0.16) | 0.80 (0.31) |
| Every day |  | 2.76 (0.30) | 2.55 (0.31) | 3.87 (0.85) |  | 2.29 (0.28) | 2.34 (0.31) | 2.08 (0.42) |

BMI: body mass index; SBP: systolic blood pressure; DBP: diastolic blood pressure; AST: aspartate aminotransferase; ALT: alanine aminotransferase. Continuous variables are represented as the mean ± standard error (SE) by complex-samples general linear models, and categorical variables are represented as the percentage (SE) by Rao-Scott chi-square tests. All statistical analyses were conducted using weight, cluster and stratification parameters to consider complex-sample survey data.

**Supplementary Table 2.** Basic characteristics of hyperlipidemia and normal groups.

| Variables | Men |  |  |  | Women |  |  |  |
| --- | --- | --- | --- | --- | --- | --- | --- | --- |
|  | P-value |  | Normal | Hyperlipidemia | P-value |  | Normal | Hyperlipidemia |
| Number of subjects |  |  | 4,066 | 367 |  |  | 5,726 | 631 |
| Age (years) | <.001 |  | 43.16 ± 0.34 | 51.18 ± 0.8 | <.001 |  | 44.24 ± 0.34 | 59.44 ± 0.53 |
| BMI (kg/m^2^) | <.001 |  | 23.91 ± 0.06 | 25.5 ± 0.2 | <.001 |  | 23.15 ± 0.07 | 25.21 ± 0.15 |
| Waist circumference (cm) | <.001 |  | 83.46 ± 0.20 | 88.88 ± 0.56 | <.001 |  | 77.40 ± 0.20 | 84.24 ± 0.44 |
| Waist-to-height ratio | <.001 |  | 0.49 ± 0.001 | 0.52 ± 0.003 | <.001 |  | 0.49 ± 0.001 | 0.55 ± 0.003 |
| SBP (mmHg) | <.001 |  | 121.20 ± 0.32 | 124.94 ± 0.96 | <.001 |  | 114.90 ± 0.33 | 127.21 ± 0.90 |
| DBP (mmHg) | .059 |  | 80.65 ± 0.25 | 82.02 ± 0.66 | <.001 |  | 74.14 ± 0.20 | 78.12 ± 0.50 |
| Pulse rate (beats per 15 seconds) | .289 |  | 17.56 ± 0.05 | 17.73 ± 0.16 | .001 |  | 17.95 ± 0.06 | 17.56 ± 0.10 |
| Hemoglobin (mg/dl) | .001 |  | 15.34 ± 0.02 | 15.08 ± 0.08 | <.001 |  | 12.95 ± 0.02 | 13.18 ± 0.05 |
| Cholesterol (mg/dl) | .003 |  | 186.69 ± 0.71 | 195.39 ± 2.81 | <.001 |  | 186.07 ± 0.61 | 198.82 ± 2.26 |
| Triglyceride (mg/dl) | <.001 |  | 151.60 ± 2.59 | 208.82 ± 9.25 | <.001 |  | 106.60 ± 1.22 | 153.64 ± 4.48 |
| Glucose (mg/dl) | <.001 |  | 96.98 ± 0.43 | 107.97 ± 1.95 | <.001 |  | 93.34 ± 0.28 | 106.39 ± 1.55 |
| AST (IU/L) | .004 |  | 24.75 ± 0.28 | 27.06 ± 0.75 | <.001 |  | 19.57 ± 0.12 | 23.15 ± 0.48 |
| ALT (IU/L) | <.001 |  | 26.81 ± 0.37 | 33.07 ± 1.49 | <.001 |  | 16.88 ± 0.20 | 21.63 ± 0.58 |
| Creatinine (mg/dl) | .133 |  | 0.95 ± 0.003 | 1.03 ± 0.05 | <.001 |  | 0.70 ± 0.002 | 0.74 ± 0.01 |
| Trunk fat mass (kg) | <.001 |  | 8.44 ± 0.08 | 10.35 ± 0.21 | <.001 |  | 9.52 ± 0.08 | 11.81 ± 0.16 |
| Percent trunk fat mass (%) | <.001 |  | 24.16 ± 0.17 | 27.76 ± 0.36 | <.001 |  | 32.82 ± 0.17 | 37.60 ± 0.28 |
| Whole-body total fat mass (kg) | <.001 |  | 15.56 ± 0.14 | 17.93 ± 0.35 | <.001 |  | 19.10 ± 0.12 | 21.31 ± 0.25 |
| Percent whole-body total fat mass (%) | <.001 |  | 22.01 ± 0.15 | 24.31 ± 0.30 | <.001 |  | 32.99 ± 0.14 | 35.39 ± 0.23 |
| Mean sleep duration (hours) | .161 |  | 6.90 ± 0.02 | 6.78 ± 0.08 | <.001 |  | 6.90 ± 0.03 | 6.56 ± 0.09 |
| Region (city) | .036 |  |  |  | .015 |  |  |  |
| Seoul |  | 21.58 (1.10) | 21.51 (1.14) | 22.43 (2.96) |  | 20.8 (0.91) | 20.46 (0.91) | 24.68 (2.40) |
| Busan |  | 7.89 (0.74) | 8.05 (0.78) | 5.80 (1.44) |  | 7.82 (0.84) | 7.58 (0.86) | 10.59 (1.53) |
| Daegu |  | 5.15 (0.71) | 5.13 (0.71) | 5.46 (1.60) |  | 5.11 (0.70) | 5.18 (0.74) | 4.34 (1.03) |
| Incheon |  | 5.30 (0.58) | 5.20 (0.57) | 6.69 (1.52) |  | 5.15 (0.52) | 5.20 (0.53) | 4.52 (1.00) |
| Gwangju |  | 2.27 (0.69) | 2.27 (0.66) | 2.25 (1.29) |  | 2.21 (0.60) | 2.28 (0.64) | 1.44 (0.47) |
| Daejeon |  | 3.18 (0.68) | 3.22 (0.69) | 2.69 (1.12) |  | 3.36 (0.68) | 3.47 (0.72) | 2.08 (0.71) |
| Ulsan |  | 2.11 (0.64) | 2.07 (0.65) | 2.65 (1.06) |  | 2.55 (0.75) | 2.55 (0.76) | 2.56 (0.98) |
| Gyeonggi-do |  | 22.85 (1.11) | 22.66 (1.12) | 25.34 (3.01) |  | 22.98 (0.97) | 23.14 (1.01) | 21.2 (1.93) |
| Gangwon-do |  | 2.34 (0.39) | 2.42 (0.40) | 1.22 (0.45) |  | 2.32 (0.50) | 2.33 (0.53) | 2.27 (0.57) |
| Chungcheongbuk-do |  | 3.36 (0.61) | 3.52 (0.65) | 1.22 (0.30) |  | 3.34 (0.57) | 3.40 (0.57) | 2.68 (0.79) |
| Chungcheongnam-do |  | 3.95 (0.66) | 3.96 (0.67) | 3.79 (1.21) |  | 4.59 (0.67) | 4.53 (0.69) | 5.21 (1.34) |
| Jeollabuk-do |  | 2.98 (0.47) | 3.02 (0.48) | 2.49 (0.59) |  | 3.06 (0.47) | 2.89 (0.46) | 4.91 (1.35) |
| Jeollanam-do |  | 2.80 (0.48) | 2.93 (0.50) | 1.12 (0.46) |  | 3.24 (0.51) | 3.41 (0.53) | 1.36 (0.44) |
| Gyeongsangbuk-do |  | 5.58 (0.72) | 5.25 (0.73) | 10.00 (2.39) |  | 5.79 (0.74) | 5.72 (0.75) | 6.66 (1.12) |
| Gyeongsangnam-do |  | 6.83 (0.95) | 6.92 (0.98) | 5.68 (1.65) |  | 5.73 (0.77) | 5.86 (0.81) | 4.19 (1.26) |
| Jeju-do |  | 1.82 (0.67) | 1.87 (0.67) | 1.17 (0.83) |  | 1.95 (0.96) | 2.00 (0.99) | 1.29 (0.62) |
| Town | .341 |  |  |  | .924 |  |  |  |
| Dong (city) |  | 80.62 (1.83) | 80.45 (1.83) | 82.84 (2.92) |  | 80.37 (1.81) | 80.39 (1.82) | 80.19 (2.58) |
| Eup, Myeon (rural) |  | 19.38 (1.83) | 19.55 (1.83) | 17.16 (2.92) |  | 19.63 (1.81) | 19.61 (1.82) | 19.81 (2.58) |
| Income | .343 |  |  |  | .094 |  |  |  |
| 1st quartile (low) |  | 25.78 (0.96) | 25.99 (0.99) | 23.02 (2.83) |  | 26.42 (0.85) | 26.8 (0.89) | 22.14 (1.96) |
| 2nd quartile (lower-middle) |  | 26.27 (0.89) | 26.49 (0.91) | 23.46 (2.74) |  | 25.37 (0.74) | 25.48 (0.79) | 24.11 (2.06) |
| 3rd quartile (upper-middle) |  | 23.33 (0.76) | 23.17 (0.79) | 25.42 (2.69) |  | 24.97 (0.73) | 24.73 (0.76) | 27.69 (2.16) |
| 4th quartile (high) |  | 24.62 (0.98) | 24.36 (1.01) | 28.10 (2.94) |  | 23.24 (0.84) | 23.00 (0.86) | 26.06 (2.22) |
| Education | .011 |  |  |  | <.001 |  |  |  |
| Elementary school or less |  | 11.98 (0.60) | 11.94 (0.63) | 12.47 (2.11) |  | 25.5 (0.89) | 23.46 (0.89) | 48.72 (2.59) |
| Middle school |  | 10.32 (0.52) | 9.89 (0.52) | 15.87 (2.29) |  | 9.50 (0.44) | 8.98 (0.44) | 15.43 (1.73) |
| High school |  | 40.35 (1.06) | 40.91 (1.10) | 32.88 (3.24) |  | 37.45 (0.96) | 38.43 (0.99) | 26.24 (2.37) |
| University or higher |  | 37.36 (1.13) | 37.25 (1.17) | 38.79 (3.25) |  | 27.56 (0.89) | 29.14 (0.93) | 9.61 (1.54) |
| Occupation | .410 |  |  |  | <.001 |  |  |  |
| Managers, professionals and related workers |  | 17.91 (0.79) | 17.83 (0.83) | 18.96 (2.63) |  | 10.23 (0.51) | 10.82 (0.55) | 3.50 (0.93) |
| Clerks |  | 11.60 (0.59) | 11.50 (0.62) | 12.94 (2.01) |  | 6.39 (0.36) | 6.88 (0.39) | 0.86 (0.44) |
| Service workers and sale workers |  | 12.84 (0.66) | 12.75 (0.67) | 13.98 (2.31) |  | 14.55 (0.62) | 14.70 (0.64) | 12.90 (1.74) |
| Skilled agricultural, forestry and fishery workers |  | 7.71 (0.89) | 7.85 (0.93) | 5.95 (1.44) |  | 5.51 (0.66) | 5.40 (0.67) | 6.68 (1.19) |
| Craft, plant, machine operators and assemblers |  | 20.09 (0.82) | 20.02 (0.87) | 21.01 (2.93) |  | 2.30 (0.23) | 2.29 (0.23) | 2.42 (0.76) |
| Elementary occupations |  | 7.25 (0.49) | 7.50 (0.52) | 3.88 (1.15) |  | 8.40 (0.44) | 8.42 (0.47) | 8.07 (1.33) |
| Unemployed |  | 22.61 (0.91) | 22.56 (0.94) | 23.28 (2.53) |  | 52.62 (0.86) | 51.48 (0.89) | 65.57 (2.40) |
| Drinking | .127 |  |  |  | <.001 |  |  |  |
| Not at all for the past one year |  | 13.01 (0.65) | 12.70 (0.67) | 17.13 (2.25) |  | 33.08 (0.84) | 31.57 (0.87) | 50.2 (2.45) |
| Less than once a month |  | 9.68 (0.61) | 9.99 (0.65) | 5.56 (1.23) |  | 26.72 (0.68) | 27.11 (0.71) | 22.23 (1.95) |
| Once a month |  | 9.23 (0.55) | 9.27 (0.57) | 8.71 (1.98) |  | 11.73 (0.49) | 11.94 (0.52) | 9.4 (1.43) |
| 2 to 4 times a month |  | 30.70 (0.87) | 30.76 (0.90) | 29.91 (2.88) |  | 19.48 (0.66) | 20.22 (0.71) | 11.11 (1.79) |
| 2 or 3 times a week |  | 24.23 (0.78) | 24.16 (0.84) | 25.18 (2.54) |  | 6.94 (0.44) | 7.07 (0.46) | 5.49 (1.36) |
| 4 or more times a week |  | 13.14 (0.62) | 13.12 (0.64) | 13.52 (2.27) |  | 2.05 (0.22) | 2.09 (0.24) | 1.57 (0.60) |
| Smoking | <.001 |  |  |  | <.001 |  |  |  |
| Smoking |  | 46.40 (0.99) | 46.75 (1.03) | 41.78 (3.26) |  | 6.58 (0.42) | 6.94 (0.45) | 2.46 (0.69) |
| Quit smoking |  | 33.40 (0.87) | 32.24 (0.90) | 48.65 (3.24) |  | 6.39 (0.41) | 6.70 (0.44) | 2.89 (0.69) |
| Never smoked |  | 20.20 (0.76) | 21.01 (0.81) | 9.58 (1.60) |  | 87.03 (0.58) | 86.36 (0.62) | 94.65 (0.99) |
| Stress | .499 |  |  |  | .243 |  |  |  |
| Extremely |  | 4.10 (0.34) | 4.24 (0.36) | 2.30 (0.85) |  | 5.26 (0.34) | 5.20 (0.35) | 6.02 (1.16) |
| Very |  | 22.36 (0.79) | 22.36 (0.83) | 22.42 (2.56) |  | 26.32 (0.72) | 26.19 (0.74) | 27.84 (2.18) |
| Slightly |  | 59.50 (0.88) | 59.38 (0.92) | 61.12 (3.09) |  | 56.18 (0.75) | 56.55 (0.77) | 51.91 (2.28) |
| Rarely |  | 14.04 (0.59) | 14.03 (0.61) | 14.16 (2.22) |  | 12.24 (0.48) | 12.06 (0.51) | 14.23 (1.50) |
| Exercise | .471 |  |  |  | .962 |  |  |  |
| Not at all |  | 55.8 (0.97) | 55.9 (0.99) | 54.48 (3.08) |  | 74.19 (0.80) | 74.13 (0.82) | 74.87 (2.20) |
| Once a week |  | 15.66 (0.75) | 15.86 (0.76) | 12.98 (2.46) |  | 6.56 (0.39) | 6.57 (0.40) | 6.50 (1.32) |
| 2 times a week |  | 9.72 (0.54) | 9.66 (0.56) | 10.62 (2.09) |  | 5.24 (0.34) | 5.29 (0.36) | 4.70 (0.96) |
| 3 times a week |  | 7.26 (0.45) | 7.22 (0.47) | 7.71 (1.67) |  | 5.74 (0.41) | 5.68 (0.42) | 6.50 (1.24) |
| 4 times a week |  | 3.19 (0.33) | 3.13 (0.33) | 3.96 (1.68) |  | 2.23 (0.26) | 2.27 (0.28) | 1.80 (0.58) |
| 5 times a week |  | 3.49 (0.34) | 3.47 (0.36) | 3.84 (1.32) |  | 2.78 (0.25) | 2.82 (0.26) | 2.34 (0.77) |
| 6 times a week |  | 2.12 (0.27) | 1.96 (0.27) | 4.18 (1.50) |  | 0.97 (0.14) | 0.99 (0.15) | 0.72 (0.36) |
| Every day |  | 2.76 (0.30) | 2.80 (0.32) | 2.24 (0.83) |  | 2.29 (0.28) | 2.27 (0.29) | 2.57 (0.81) |

BMI: body mass index; SBP: systolic blood pressure; DBP: diastolic blood pressure; AST: aspartate aminotransferase; ALT: alanine aminotransferase. Continuous variables are represented as the mean ± standard error (SE) by complex-samples general linear models, and categorical variables are represented as the percentage (SE) by Rao-Scott chi-square tests. All statistical analyses were conducted using weight, cluster and stratification parameters to consider complex-sample survey data.

**Supplementary Table 3.** Basic characteristics of diabetes and normal groups.

| Variables | Men |  |  |  | Women |  |  |  |
| --- | --- | --- | --- | --- | --- | --- | --- | --- |
|  | P-value |  | Normal | Diabetes | P-value |  | Normal | Diabetes |
| Number of subjects |  |  | 4,062 | 371 |  |  | 5,913 | 444 |
| Age (years) | <.001 |  | 42.80 ± 0.33 | 57.98 ± 0.66 | <.001 |  | 44.47 ± 0.33 | 61.93 ± 0.82 |
| BMI (kg/m^2^) | <.001 |  | 23.96 ± 0.06 | 24.99 ± 0.23 | <.001 |  | 23.19 ± 0.07 | 25.38 ± 0.19 |
| Waist circumference (cm) | <.001 |  | 83.50 ± 0.19 | 89.00 ± 0.60 | <.001 |  | 77.45 ± 0.20 | 86.25 ± 0.57 |
| Waist-to-height ratio | <.001 |  | 0.49 ± 0.001 | 0.53 ± 0.004 | <.001 |  | 0.49 ± 0.001 | 0.56 ± 0.004 |
| SBP (mmHg) | <.001 |  | 121.08 ± 0.31 | 127.28 ± 1.27 | <.001 |  | 115.05 ± 0.33 | 129.81 ± 1.00 |
| DBP (mmHg) | .287 |  | 80.79 ± 0.24 | 80.07 ± 0.66 | <.001 |  | 74.32 ± 0.21 | 76.79 ± 0.56 |
| Pulse rate (beats per 15 seconds) | <.001 |  | 17.51 ± 0.05 | 18.44 ± 0.19 | .003 |  | 17.89 ± 0.05 | 18.33 ± 0.15 |
| Hemoglobin (mg/dl) | <.001 |  | 15.36 ± 0.02 | 14.80 ± 0.10 | .602 |  | 12.97 ± 0.02 | 13.00 ± 0.07 |
| Cholesterol (mg/dl) | <.001 |  | 187.92 ± 0.70 | 177.87 ± 2.36 | .071 |  | 186.86 ± 0.62 | 191.03 ± 2.25 |
| Triglyceride (mg/dl) | .013 |  | 154.29 ± 2.53 | 176.33 ± 8.49 | <.001 |  | 107.07 ± 1.25 | 164.93 ± 5.90 |
| Glucose (mg/dl) | <.001 |  | 94.97 ± 0.35 | 140.6 ± 3.37 | <.001 |  | 91.83 ± 0.20 | 136.47 ± 2.68 |
| AST (IU/L) | .002 |  | 24.73 ± 0.28 | 27.83 ± 0.96 | <.001 |  | 19.63 ± 0.12 | 23.67 ± 0.70 |
| ALT (IU/L) | .010 |  | 27.06 ± 0.38 | 30.24 ± 1.17 | <.001 |  | 16.95 ± 0.20 | 22.4 ± 0.77 |
| Creatinine (mg/dl) | .062 |  | 0.95 ± 0.005 | 0.99 ± 0.02 | <.001 |  | 0.7 ± 0.002 | 0.76 ± 0.01 |
| Trunk fat mass (kg) | <.001 |  | 8.49 ± 0.08 | 9.85 ± 0.24 | <.001 |  | 9.57 ± 0.07 | 11.88 ± 0.21 |
| Percent trunk fat mass (%) | <.001 |  | 24.25 ± 0.17 | 27.00 ± 0.42 | <.001 |  | 32.97 ± 0.17 | 37.12 ± 0.38 |
| Whole-body total fat mass (kg) | .005 |  | 15.66 ± 0.14 | 16.75 ± 0.37 | <.001 |  | 19.18 ± 0.12 | 20.87 ± 0.32 |
| Percent whole-body total fat mass (%) | <.001 |  | 22.08 ± 0.15 | 23.64 ± 0.36 | <.001 |  | 33.10 ± 0.14 | 34.62 ± 0.31 |
| Mean sleep duration (hours) | .883 |  | 6.89 ± 0.02 | 6.91 ± 0.10 | .006 |  | 6.89 ± 0.02 | 6.59 ± 0.11 |
| Region (city) | .001 |  |  |  | .021 |  |  |  |
| Seoul |  | 21.58 (1.10) | 21.79 (1.14) | 18.29 (2.52) |  | 20.8 (0.91) | 20.93 (0.93) | 18.62 (2.71) |
| Busan |  | 7.89 (0.74) | 8.07 (0.76) | 5.13 (1.19) |  | 7.82 (0.84) | 7.54 (0.84) | 12.41 (1.99) |
| Daegu |  | 5.15 (0.71) | 5.14 (0.69) | 5.30 (1.64) |  | 5.11 (0.70) | 5.19 (0.71) | 3.84 (1.35) |
| Incheon |  | 5.30 (0.58) | 5.24 (0.60) | 6.24 (1.77) |  | 5.15 (0.52) | 5.22 (0.53) | 3.86 (1.11) |
| Gwangju |  | 2.27 (0.69) | 2.33 (0.71) | 1.32 (0.60) |  | 2.21 (0.60) | 2.26 (0.63) | 1.41 (0.60) |
| Daejeon |  | 3.18 (0.68) | 3.26 (0.70) | 1.96 (0.72) |  | 3.36 (0.68) | 3.38 (0.7) | 3.05 (1.01) |
| Ulsan |  | 2.11 (0.64) | 1.85 (0.58) | 6.06 (2.61) |  | 2.55 (0.75) | 2.60 (0.77) | 1.76 (0.83) |
| Gyeonggi-do |  | 22.85 (1.11) | 23.02 (1.16) | 20.31 (2.86) |  | 22.98 (0.97) | 23.14 (1.01) | 20.4 (2.77) |
| Gangwon-do |  | 2.34 (0.39) | 2.29 (0.39) | 3.06 (0.70) |  | 2.32 (0.50) | 2.26 (0.50) | 3.29 (1.31) |
| Chungcheongbuk-do |  | 3.36 (0.61) | 3.33 (0.63) | 3.80 (1.02) |  | 3.34 (0.57) | 3.43 (0.59) | 1.80 (0.60) |
| Chungcheongnam-do |  | 3.95 (0.66) | 3.96 (0.68) | 3.77 (1.24) |  | 4.59 (0.67) | 4.54 (0.68) | 5.30 (1.67) |
| Jeollabuk-do |  | 2.98 (0.47) | 2.80 (0.50) | 5.76 (1.32) |  | 3.06 (0.47) | 2.86 (0.44) | 6.21 (1.81) |
| Jeollanam-do |  | 2.80 (0.48) | 2.73 (0.49) | 3.96 (0.94) |  | 3.24 (0.51) | 3.20 (0.51) | 3.96 (1.03) |
| Gyeongsangbuk-do |  | 5.58 (0.72) | 5.41 (0.74) | 8.23 (2.36) |  | 5.79 (0.74) | 5.75 (0.77) | 6.45 (1.58) |
| Gyeongsangnam-do |  | 6.83 (0.95) | 6.95 (0.97) | 5.09 (1.82) |  | 5.73 (0.77) | 5.70 (0.79) | 6.17 (1.29) |
| Jeju-do |  | 1.82 (0.67) | 1.83 (0.66) | 1.72 (1.01) |  | 1.95 (0.96) | 1.98 (0.98) | 1.47 (0.60) |
| Town | .024 |  |  |  | .050 |  |  |  |
| Dong (city) |  | 80.62 (1.83) | 81.03 (1.81) | 74.35 (3.73) |  | 80.37 (1.81) | 80.66 (1.81) | 75.64 (3.21) |
| Eup, Myeon (rural) |  | 19.38 (1.83) | 18.97 (1.81) | 25.65 (3.73) |  | 19.63 (1.81) | 19.34 (1.81) | 24.36 (3.21) |
| Income | .013 |  |  |  | .721 |  |  |  |
| 1st quartile (low) |  | 25.78 (0.96) | 25.21 (0.99) | 34.45 (3.49) |  | 26.42 (0.85) | 26.58 (0.87) | 23.87 (2.57) |
| 2nd quartile (lower-middle) |  | 26.27 (0.89) | 26.35 (0.92) | 25.07 (2.69) |  | 25.37 (0.74) | 25.28 (0.77) | 26.83 (2.43) |
| 3rd quartile (upper-middle) |  | 23.33 (0.76) | 23.69 (0.80) | 17.70 (2.38) |  | 24.97 (0.73) | 24.88 (0.75) | 26.39 (2.49) |
| 4th quartile (high) |  | 24.62 (0.98) | 24.74 (1.03) | 22.78 (2.63) |  | 23.24 (0.84) | 23.26 (0.86) | 22.91 (2.50) |
| Education | <.001 |  |  |  | <.001 |  |  |  |
| Elementary school or less |  | 11.98 (0.60) | 10.92 (0.58) | 28.31 (3.02) |  | 25.5 (0.89) | 23.14 (0.87) | 64.12 (2.86) |
| Middle school |  | 10.32 (0.52) | 9.68 (0.52) | 20.03 (2.47) |  | 9.50 (0.44) | 9.52 (0.46) | 9.17 (1.56) |
| High school |  | 40.35 (1.06) | 40.69 (1.11) | 35.11 (3.35) |  | 37.45 (0.96) | 38.44 (0.98) | 21.11 (2.51) |
| University or higher |  | 37.36 (1.13) | 38.71 (1.18) | 16.56 (2.48) |  | 27.56 (0.89) | 28.9 (0.92) | 5.61 (1.49) |
| Occupation | <.001 |  |  |  | <.001 |  |  |  |
| Managers, professionals and related workers |  | 17.91 (0.79) | 18.46 (0.82) | 9.45 (1.87) |  | 10.23 (0.51) | 10.76 (0.54) | 1.6 (0.85) |
| Clerks |  | 11.60 (0.59) | 12.02 (0.62) | 5.18 (1.38) |  | 6.39 (0.36) | 6.74 (0.38) | 0.76 (0.39) |
| Service workers and sale workers |  | 12.84 (0.66) | 12.92 (0.66) | 11.49 (2.21) |  | 14.55 (0.62) | 14.96 (0.64) | 7.89 (1.67) |
| Skilled agricultural, forestry and fishery workers |  | 7.71 (0.89) | 7.42 (0.88) | 12.23 (2.32) |  | 5.51 (0.66) | 5.18 (0.65) | 10.9 (2.00) |
| Craft, plant, machine operators and assemblers |  | 20.09 (0.82) | 20.30 (0.88) | 16.95 (2.66) |  | 2.30 (0.23) | 2.32 (0.23) | 2.08 (0.85) |
| Elementary occupations |  | 7.25 (0.49) | 6.96 (0.49) | 11.68 (2.27) |  | 8.40 (0.44) | 8.26 (0.46) | 10.66 (1.91) |
| Unemployed |  | 22.61 (0.91) | 21.93 (0.94) | 33.02 (3.09) |  | 52.62 (0.86) | 51.8 (0.88) | 66.11 (3.15) |
| Drinking | <.001 |  |  |  | <.001 |  |  |  |
| Not at all for the past one year |  | 13.01 (0.65) | 12.39 (0.66) | 22.58 (2.43) |  | 33.08 (0.84) | 31.55 (0.84) | 58.07 (3.09) |
| Less than once a month |  | 9.68 (0.61) | 9.55 (0.63) | 11.70 (2.20) |  | 26.72 (0.68) | 27.21 (0.71) | 18.67 (2.41) |
| Once a month |  | 9.23 (0.55) | 9.37 (0.57) | 7.10 (1.44) |  | 11.73 (0.49) | 11.93 (0.51) | 8.54 (1.63) |
| 2 to 4 times a month |  | 30.70 (0.87) | 31.33 (0.89) | 21.15 (2.62) |  | 19.48 (0.66) | 20.09 (0.69) | 9.62 (1.95) |
| 2 or 3 times a week |  | 24.23 (0.78) | 24.69 (0.82) | 17.22 (2.48) |  | 6.94 (0.44) | 7.13 (0.45) | 3.82 (1.28) |
| 4 or more times a week |  | 13.14 (0.62) | 12.68 (0.63) | 20.26 (3.12) |  | 2.05 (0.22) | 2.09 (0.23) | 1.27 (0.84) |
| Smoking | <.001 |  |  |  | .582 |  |  |  |
| Smoking |  | 46.40 (0.99) | 46.65 (1.01) | 42.63 (3.20) |  | 6.58 (0.42) | 6.63 (0.44) | 5.68 (1.44) |
| Quit smoking |  | 33.40 (0.87) | 32.66 (0.91) | 44.74 (2.78) |  | 6.39 (0.41) | 6.46 (0.44) | 5.28 (1.22) |
| Never smoked |  | 20.20 (0.76) | 20.69 (0.80) | 12.63 (2.03) |  | 87.03 (0.58) | 86.91 (0.61) | 89.04 (1.80) |
| Stress | .014 |  |  |  | <.001 |  |  |  |
| Extremely |  | 4.10 (0.34) | 4.05 (0.35) | 4.84 (1.49) |  | 5.26 (0.34) | 5.15 (0.35) | 7.09 (1.37) |
| Very |  | 22.36 (0.79) | 22.74 (0.83) | 16.52 (2.54) |  | 26.32 (0.72) | 26.33 (0.74) | 26.20 (2.56) |
| Slightly |  | 59.50 (0.88) | 59.58 (0.92) | 58.27 (2.99) |  | 56.18 (0.75) | 57.01 (0.76) | 42.46 (2.97) |
| Rarely |  | 14.04 (0.59) | 13.63 (0.60) | 20.38 (2.50) |  | 12.24 (0.48) | 11.51 (0.48) | 24.26 (2.55) |
| Exercise | <.001 |  |  |  | .274 |  |  |  |
| Not at all |  | 55.80 (0.97) | 54.87 (1.01) | 70.10 (3.04) |  | 74.19 (0.80) | 73.89 (0.84) | 79.01 (2.94) |
| Once a week |  | 15.66 (0.75) | 16.29 (0.78) | 6.02 (1.67) |  | 6.56 (0.39) | 6.66 (0.40) | 4.97 (1.68) |
| 2 times a week |  | 9.72 (0.54) | 10.01 (0.57) | 5.36 (1.35) |  | 5.24 (0.34) | 5.29 (0.35) | 4.32 (1.17) |
| 3 times a week |  | 7.26 (0.45) | 7.14 (0.47) | 9.02 (2.31) |  | 5.74 (0.41) | 5.83 (0.43) | 4.38 (1.49) |
| 4 times a week |  | 3.19 (0.33) | 3.24 (0.34) | 2.31 (0.84) |  | 2.23 (0.26) | 2.29 (0.27) | 1.28 (0.57) |
| 5 times a week |  | 3.49 (0.34) | 3.53 (0.36) | 2.92 (1.19) |  | 2.78 (0.25) | 2.82 (0.26) | 2.19 (0.74) |
| 6 times a week |  | 2.12 (0.27) | 2.23 (0.29) | 0.39 (0.28) |  | 0.97 (0.14) | 1.02 (0.15) | 0.17 (0.17) |
| Every day |  | 2.76 (0.30) | 2.69 (0.31) | 3.88 (1.26) |  | 2.29 (0.28) | 2.21 (0.28) | 3.67 (1.37) |

BMI: body mass index; SBP: systolic blood pressure; DBP: diastolic blood pressure; AST: aspartate aminotransferase; ALT: alanine aminotransferase. Continuous variables are represented as the mean ± standard error (SE) by complex-samples general linear models, and categorical variables are represented as the percentage (SE) by Rao-Scott chi-square tests. All statistical analyses were conducted using weight, cluster and stratification parameters to consider complex-sample survey data.

**Supplementary Table 4.** Basic characteristics of hypercholesterolemia and normal groups.

| Variables | Men |  |  |  | Women |  |  |  |
| --- | --- | --- | --- | --- | --- | --- | --- | --- |
|  | P-value |  | Normal | Hypercholesterolemia | P-value |  | Normal | Hypercholesterolemia |
| Number of subjects |  |  | 3,908 | 525 |  |  | 5,440 | 917 |
| Age (years) | <.001 |  | 43.09 ± 0.35 | 49.01 ± 0.63 | <.001 |  | 43.86 ± 0.34 | 57.04 ± 0.55 |
| BMI (kg/m^2^) | <.001 |  | 23.88 ± 0.06 | 25.23 ± 0.18 | <.001 |  | 23.07 ± 0.07 | 25.06 ± 0.13 |
| Waist circumference (cm) | <.001 |  | 83.34 ± 0.20 | 87.95 ± 0.48 | <.001 |  | 77.14 ± 0.21 | 83.78 ± 0.41 |
| Waist-to-height ratio | <.001 |  | 0.49 ± 0.001 | 0.52 ± 0.003 | <.001 |  | 0.49 ± 0.001 | 0.54 ± 0.003 |
| SBP (mmHg) | <.001 |  | 120.91 ± 0.31 | 125.99 ± 0.95 | <.001 |  | 114.36 ± 0.33 | 126.91 ± 0.77 |
| DBP (mmHg) | <.001 |  | 80.43 ± 0.24 | 83.38 ± 0.60 | <.001 |  | 73.81 ± 0.20 | 79.12 ± 0.43 |
| Pulse rate (beats per 15 seconds) | .006 |  | 17.53 ± 0.05 | 17.9 ± 0.13 | .030 |  | 17.95 ± 0.06 | 17.73 ± 0.10 |
| Hemoglobin (mg/dl) | .254 |  | 15.32 ± 0.02 | 15.39 ± 0.06 | <.001 |  | 12.93 ± 0.02 | 13.28 ± 0.04 |
| Cholesterol (mg/dl) | <.001 |  | 181.52 ± 0.59 | 235.25 ± 2.55 | <.001 |  | 181.17 ± 0.50 | 229.68 ± 2.14 |
| Triglyceride (mg/dl) | <.001 |  | 143.82 ± 2.05 | 253.52 ± 14.13 | <.001 |  | 103.71 ± 1.17 | 158.40 ± 4.15 |
| Glucose (mg/dl) | <.001 |  | 96.5 ± 0.39 | 108.17 ± 2.10 | <.001 |  | 92.88 ± 0.27 | 105.26 ± 1.23 |
| AST (IU/L) | .002 |  | 24.6 ± 0.29 | 27.56 ± 0.89 | <.001 |  | 19.41 ± 0.13 | 23.05 ± 0.41 |
| ALT (IU/L) | <.001 |  | 26.37 ± 0.37 | 34.61 ± 1.21 | <.001 |  | 16.59 ± 0.20 | 22.08 ± 0.61 |
| Creatinine (mg/dl) | .320 |  | 0.96 ± 0.005 | 0.97 ± 0.01 | <.001 |  | 0.7 ± 0.002 | 0.73 ± 0.01 |
| Trunk fat mass (kg) | <.001 |  | 8.38 ± 0.08 | 10.17 ± 0.19 | <.001 |  | 9.44 ± 0.08 | 11.62 ± 0.15 |
| Percent trunk fat mass (%) | <.001 |  | 24.02 ± 0.18 | 27.70 ± 0.32 | <.001 |  | 32.64 ± 0.18 | 37.24 ± 0.26 |
| Whole-body total fat mass (kg) | <.001 |  | 15.49 ± 0.14 | 17.71 ± 0.31 | <.001 |  | 18.99 ± 0.12 | 21.31 ± 0.23 |
| Percent whole-body total fat mass (%) | <.001 |  | 21.90 ± 0.15 | 24.39 ± 0.27 | <.001 |  | 32.88 ± 0.142 | 35.42 ± 0.22 |
| Mean sleep duration (hours) | .515 |  | 6.90 ± 0.02 | 6.85 ± 0.07 | .005 |  | 6.89 ± 0.03 | 6.70 ± 0.07 |
| Region (city) | .436 |  |  |  | .293 |  |  |  |
| Seoul |  | 21.58 (1.10) | 21.89 (1.17) | 18.95 (2.06) |  | 20.8 (0.91) | 20.66 (0.96) | 21.80 (1.86) |
| Busan |  | 7.89 (0.74) | 7.75 (0.80) | 9.10 (1.51) |  | 7.82 (0.84) | 7.81 (0.91) | 7.89 (1.07) |
| Daegu |  | 5.15 (0.71) | 5.19 (0.71) | 4.79 (1.30) |  | 5.11 (0.70) | 5.31 (0.74) | 3.69 (0.80) |
| Incheon |  | 5.30 (0.58) | 5.11 (0.56) | 6.90 (1.46) |  | 5.15 (0.52) | 5.27 (0.55) | 4.23 (1.02) |
| Gwangju |  | 2.27 (0.69) | 2.25 (0.66) | 2.42 (1.19) |  | 2.21 (0.60) | 2.26 (0.63) | 1.87 (0.66) |
| Daejeon |  | 3.18 (0.68) | 3.29 (0.71) | 2.31 (0.85) |  | 3.36 (0.68) | 3.55 (0.75) | 1.97 (0.79) |
| Ulsan |  | 2.11 (0.64) | 1.91 (0.61) | 3.77 (1.73) |  | 2.55 (0.75) | 2.64 (0.80) | 1.88 (0.75) |
| Gyeonggi-do |  | 22.85 (1.11) | 22.89 (1.17) | 22.55 (2.21) |  | 22.98 (0.97) | 22.88 (1.05) | 23.72 (1.79) |
| Gangwon-do |  | 2.34 (0.39) | 2.32 (0.40) | 2.48 (0.82) |  | 2.32 (0.50) | 2.33 (0.54) | 2.31 (0.54) |
| Chungcheongbuk-do |  | 3.36 (0.61) | 3.59 (0.68) | 1.44 (0.36) |  | 3.34 (0.57) | 3.27 (0.55) | 3.86 (1.03) |
| Chungcheongnam-do |  | 3.95 (0.66) | 3.88 (0.71) | 4.51 (1.34) |  | 4.59 (0.67) | 4.55 (0.68) | 4.88 (1.05) |
| Jeollabuk-do |  | 2.98 (0.47) | 2.97 (0.52) | 3.06 (0.69) |  | 3.06 (0.47) | 2.86 (0.42) | 4.48 (1.08) |
| Jeollanam-do |  | 2.80 (0.48) | 2.82 (0.49) | 2.70 (0.78) |  | 3.24 (0.51) | 3.22 (0.53) | 3.37 (0.75) |
| Gyeongsangbuk-do |  | 5.58 (0.72) | 5.48 (0.81) | 6.42 (1.68) |  | 5.79 (0.74) | 5.71 (0.77) | 6.38 (0.86) |
| Gyeongsangnam-do |  | 6.83 (0.95) | 6.90 (0.96) | 6.25 (1.67) |  | 5.73 (0.77) | 5.62 (0.79) | 6.50 (1.35) |
| Jeju-do |  | 1.82 (0.67) | 1.75 (0.62) | 2.37 (1.14) |  | 1.95 (0.96) | 2.05 (0.98) | 1.19 (0.82) |
| Town | .261 |  |  |  | .016 |  |  |  |
| Dong (city) |  | 80.62 (1.83) | 80.31 (1.87) | 83.19 (2.70) |  | 80.37 (1.81) | 80.91 (1.80) | 76.51 (2.56) |
| Eup, Myeon (rural) |  | 19.38 (1.83) | 19.69 (1.87) | 16.81 (2.70) |  | 19.63 (1.81) | 19.09 (1.80) | 23.49 (2.56) |
| Income | .314 |  |  |  | .428 |  |  |  |
| 1st quartile (low) |  | 25.78 (0.96) | 25.57 (1.00) | 27.51 (2.42) |  | 26.42 (0.85) | 26.27 (0.90) | 27.52 (1.87) |
| 2nd quartile (lower-middle) |  | 26.27 (0.89) | 26.58 (0.94) | 23.74 (2.17) |  | 25.37 (0.74) | 25.76 (0.79) | 22.59 (1.71) |
| 3rd quartile (upper-middle) |  | 23.33 (0.76) | 23.57 (0.81) | 21.33 (2.11) |  | 24.97 (0.73) | 24.86 (0.77) | 25.69 (1.77) |
| 4th quartile (high) |  | 24.62 (0.98) | 24.28 (1.04) | 27.42 (2.26) |  | 23.24 (0.84) | 23.11 (0.85) | 24.20 (1.93) |
| Education | .025 |  |  |  | <.001 |  |  |  |
| Elementary school or less |  | 11.98 (0.60) | 11.68 (0.64) | 14.51 (1.89) |  | 25.50 (0.89) | 22.47 (0.87) | 47.24 (2.14) |
| Middle school |  | 10.32 (0.52) | 9.87 (0.54) | 14.04 (1.74) |  | 9.50 (0.44) | 8.83 (0.46) | 14.26 (1.41) |
| High school |  | 40.35 (1.06) | 40.86 (1.12) | 36.12 (2.59) |  | 37.45 (0.96) | 39.13 (1.00) | 25.37 (2.04) |
| University or higher |  | 37.36 (1.13) | 37.60 (1.20) | 35.33 (2.74) |  | 27.56 (0.89) | 29.57 (0.95) | 13.13 (1.53) |
| Occupation | .255 |  |  |  | <.001 |  |  |  |
| Managers, professionals and related workers |  | 17.91 (0.79) | 17.77 (0.85) | 18.99 (2.22) |  | 10.23 (0.51) | 10.97 (0.57) | 4.9 (0.87) |
| Clerks |  | 11.60 (0.59) | 11.79 (0.63) | 10.03 (1.43) |  | 6.39 (0.36) | 6.98 (0.41) | 2.15 (0.54) |
| Service workers and sale workers |  | 12.84 (0.66) | 12.63 (0.69) | 14.54 (1.83) |  | 14.55 (0.62) | 14.89 (0.67) | 12.16 (1.23) |
| Skilled agricultural, forestry and fishery workers |  | 7.71 (0.89) | 7.67 (0.92) | 8.09 (1.64) |  | 5.51 (0.66) | 5.28 (0.67) | 7.15 (1.14) |
| Craft, plant, machine operators and assemblers |  | 20.09 (0.82) | 19.98 (0.89) | 21.07 (2.20) |  | 2.30 (0.23) | 2.27 (0.24) | 2.52 (0.62) |
| Elementary occupations |  | 7.25 (0.49) | 7.64 (0.54) | 3.99 (1.09) |  | 8.40 (0.44) | 8.28 (0.50) | 9.21 (1.17) |
| Unemployed |  | 22.61 (0.91) | 22.53 (0.97) | 23.28 (2.12) |  | 52.62 (0.86) | 51.33 (0.92) | 61.91 (2.05) |
| Drinking | <.001 |  |  |  | <.001 |  |  |  |
| Not at all for the past one year |  | 13.01 (0.65) | 12.47 (0.70) | 17.48 (1.79) |  | 33.08 (0.84) | 31.13 (0.88) | 47.08 (2.04) |
| Less than once a month |  | 9.68 (0.61) | 9.79 (0.65) | 8.75 (1.56) |  | 26.72 (0.68) | 27.45 (0.73) | 21.47 (1.58) |
| Once a month |  | 9.23 (0.55) | 9.34 (0.59) | 8.26 (1.56) |  | 11.73 (0.49) | 11.96 (0.54) | 10.14 (1.22) |
| 2 to 4 times a month |  | 30.70 (0.87) | 31.84 (0.93) | 21.31 (2.16) |  | 19.48 (0.66) | 20.10 (0.71) | 15.05 (1.52) |
| 2 or 3 times a week |  | 24.23 (0.78) | 23.80 (0.84) | 27.82 (2.22) |  | 6.94 (0.44) | 7.26 (0.47) | 4.66 (0.99) |
| 4 or more times a week |  | 13.14 (0.62) | 12.75 (0.61) | 16.39 (2.12) |  | 2.05 (0.22) | 2.11 (0.24) | 1.59 (0.45) |
| Smoking | <.001 |  |  |  | .012 |  |  |  |
| Smoking |  | 46.40 (0.99) | 46.65 (1.04) | 44.38 (2.44) |  | 6.58 (0.42) | 6.83 (0.46) | 4.75 (0.88) |
| Quit smoking |  | 33.40 (0.87) | 32.26 (0.90) | 42.85 (2.53) |  | 6.39 (0.41) | 6.67 (0.46) | 4.38 (0.81) |
| Never smoked |  | 20.20 (0.76) | 21.1 (0.82) | 12.77 (1.65) |  | 87.03 (0.58) | 86.49 (0.63) | 90.87 (1.13) |
| Stress | .388 |  |  |  | .024 |  |  |  |
| Extremely |  | 4.10 (0.34) | 4.14 (0.36) | 3.77 (1.03) |  | 5.26 (0.34) | 5.06 (0.36) | 6.73 (0.99) |
| Very |  | 22.36 (0.79) | 22.72 (0.85) | 19.42 (2.01) |  | 26.32 (0.72) | 26.32 (0.76) | 26.34 (1.70) |
| Slightly |  | 59.50 (0.88) | 59.39 (0.94) | 60.40 (2.68) |  | 56.18 (0.75) | 56.74 (0.80) | 52.11 (1.90) |
| Rarely |  | 14.04 (0.59) | 13.75 (0.61) | 16.42 (2.25) |  | 12.24 (0.48) | 11.88 (0.52) | 14.83 (1.29) |
| Exercise | .688 |  |  |  | .293 |  |  |  |
| Not at all |  | 55.8 (0.97) | 55.99 (1.00) | 54.24 (2.67) |  | 74.19 (0.80) | 73.69 (0.85) | 77.75 (1.68) |
| Once a week |  | 15.66 (0.75) | 15.75 (0.78) | 14.86 (1.93) |  | 6.56 (0.39) | 6.79 (0.42) | 4.91 (0.92) |
| 2 times a week |  | 9.72 (0.54) | 9.53 (0.57) | 11.35 (1.72) |  | 5.24 (0.34) | 5.20 (0.37) | 5.50 (0.92) |
| 3 times a week |  | 7.26 (0.45) | 7.27 (0.49) | 7.13 (1.32) |  | 5.74 (0.41) | 5.82 (0.45) | 5.19 (0.87) |
| 4 times a week |  | 3.19 (0.33) | 3.23 (0.34) | 2.80 (1.24) |  | 2.23 (0.26) | 2.33 (0.29) | 1.48 (0.40) |
| 5 times a week |  | 3.49 (0.34) | 3.46 (0.37) | 3.79 (0.91) |  | 2.78 (0.25) | 2.85 (0.27) | 2.28 (0.63) |
| 6 times a week |  | 2.12 (0.27) | 1.96 (0.28) | 3.45 (1.03) |  | 0.97 (0.14) | 1.03 (0.16) | 0.54 (0.25) |
| Every day |  | 2.76 (0.30) | 2.81 (0.32) | 2.37 (0.79) |  | 2.29 (0.28) | 2.29 (0.31) | 2.36 (0.62) |

BMI: body mass index; SBP: systolic blood pressure; DBP: diastolic blood pressure; AST: aspartate aminotransferase; ALT: alanine aminotransferase. Continuous variables are represented as the mean ± standard error (SE) by complex-samples general linear models, and categorical variables are represented as the percentage (SE) by Rao-Scott chi-square tests. All statistical analyses were conducted using weight, cluster and stratification parameters to consider complex-sample survey data.

**Supplementary Table 5.** Basic characteristics of hypo-HDL cholesterolemia and normal groups.

| Variables | Men |  |  |  | Women |  |  |  |
| --- | --- | --- | --- | --- | --- | --- | --- | --- |
|  | P-value |  | Normal | Hypo-HDL cholesterolemia | P-value |  | Normal | Hypo-HDL cholesterolemia |
| Number of subjects |  |  | 2,963 | 1,470 |  |  | 5,220 | 1,137 |
| Age (years) | <.001 |  | 42.52 ± 0.39 | 46.42 ± 0.49 | <.001 |  | 44.25 ± 0.34 | 51.53 ± 0.65 |
| BMI (kg/m^2^) | <.001 |  | 23.60 ± 0.08 | 24.97 ± 0.10 | <.001 |  | 23.04 ± 0.07 | 24.68 ± 0.15 |
| Waist circumference (cm) | <.001 |  | 82.51 ± 0.23 | 86.81 ± 0.27 | <.001 |  | 77.03 ± 0.20 | 82.55 ± 0.42 |
| Waist to height ratio | <.001 |  | 0.48 ± 0.001 | 0.51 ± 0.002 | <.001 |  | 0.49 ± 0.001 | 0.53 ± 0.003 |
| SBP (mmHg) | .114 |  | 121.16 ± 0.38 | 122.12 ± 0.49 | <.001 |  | 115.01 ± 0.35 | 120.30 ± 0.71 |
| DBP (mmHg) | .508 |  | 80.66 ± 0.28 | 80.95 ± 0.37 | <.001 |  | 74.18 ± 0.22 | 75.86 ± 0.39 |
| Pulse rate (beats per 15 seconds) | .645 |  | 17.55 ± 0.05 | 17.60 ± 0.09 | .859 |  | 17.92 ± 0.06 | 17.9 ± 0.08 |
| Hemoglobin (mg/dl) | .245 |  | 15.34 ± 0.02 | 15.29 ± 0.04 | .520 |  | 12.98 ± 0.02 | 12.95 ± 0.04 |
| Cholesterol (mg/dl) | <.001 |  | 189.38 ± 0.77 | 182.69 ± 1.27 | <.001 |  | 188.46 ± 0.68 | 180.36 ± 1.34 |
| Triglyceride (mg/dl) | <.001 |  | 132.28 ± 2.34 | 207.69 ± 5.61 | <.001 |  | 98.52 ± 1.10 | 169.58 ± 3.73 |
| Glucose (mg/dl) | <.001 |  | 96.47 ± 0.46 | 100.63 ± 0.91 | <.001 |  | 93.33 ± 0.28 | 99.67 ± 0.95 |
| AST (IU/L) | .059 |  | 24.53 ± 0.31 | 25.78 ± 0.57 | <.001 |  | 19.64 ± 0.12 | 20.93 ± 0.31 |
| ALT (IU/L) | <.001 |  | 25.75 ± 0.41 | 30.61 ± 0.75 | <.001 |  | 16.76 ± 0.19 | 19.74 ± 0.55 |
| Creatinine (mg/dl) | <.001 |  | 0.94 ± 0.003 | 0.99 ± 0.01 | <.001 |  | 0.70 ± 0.002 | 0.72 ± 0.01 |
| Trunk fat mass (kg) | <.001 |  | 8.09 ± 0.09 | 9.66 ± 0.11 | <.001 |  | 9.42 ± 0.08 | 11.1 ± 0.15 |
| Percent trunk fat mass (%) | <.001 |  | 23.46 ± 0.19 | 26.55 ± 0.22 | <.001 |  | 32.68 ± 0.17 | 35.83 ± 0.28 |
| Whole-body total fat mass (kg) | <.001 |  | 15.01 ± 0.15 | 17.33 ± 0.18 | <.001 |  | 18.97 ± 0.12 | 20.81 ± 0.23 |
| Percent whole-body total fat mass (%) | <.001 |  | 21.44 ± 0.16 | 23.80 ± 0.18 | <.001 |  | 32.93 ± 0.14 | 34.45 ± 0.21 |
| Mean sleep duration (hours) | .160 |  | 6.87 ± 0.03 | 6.94 ± 0.04 | .135 |  | 6.88 ± 0.03 | 6.81 ± 0.05 |
| Region (city) | .536 |  |  |  | .148 |  |  |  |
| Seoul |  | 21.58 (1.10) | 21.70 (1.30) | 21.30 (1.59) |  | 20.8 (0.91) | 21.31 (0.96) | 18.26 (1.86) |
| Busan |  | 7.89 (0.74) | 7.58 (0.78) | 8.58 (1.37) |  | 7.82 (0.84) | 7.71 (0.79) | 8.35 (1.57) |
| Daegu |  | 5.15 (0.71) | 4.97 (0.81) | 5.54 (1.04) |  | 5.11 (0.7) | 5.23 (0.73) | 4.53 (0.89) |
| Incheon |  | 5.30 (0.58) | 5.14 (0.65) | 5.68 (0.74) |  | 5.15 (0.52) | 5.31 (0.54) | 4.33 (0.88) |
| Gwangju |  | 2.27 (0.69) | 2.40 (0.73) | 1.97 (0.75) |  | 2.21 (0.60) | 2.24 (0.64) | 2.05 (0.62) |
| Daejeon |  | 3.18 (0.68) | 3.68 (0.79) | 2.09 (0.52) |  | 3.36 (0.68) | 3.31 (0.67) | 3.60 (0.92) |
| Ulsan |  | 2.11 (0.64) | 2.43 (0.76) | 1.41 (0.52) |  | 2.55 (0.75) | 2.40 (0.72) | 3.27 (1.22) |
| Gyeonggi-do |  | 22.85 (1.11) | 22.69 (1.31) | 23.22 (1.52) |  | 22.98 (0.97) | 23.16 (1.04) | 22.1 (1.99) |
| Gangwon-do |  | 2.34 (0.39) | 2.51 (0.44) | 1.95 (0.48) |  | 2.32 (0.50) | 2.22 (0.48) | 2.85 (0.73) |
| Chungcheongbuk-do |  | 3.36 (0.61) | 3.53 (0.67) | 2.98 (0.58) |  | 3.34 (0.57) | 3.37 (0.62) | 3.22 (0.68) |
| Chungcheongnam-do |  | 3.95 (0.66) | 3.87 (0.77) | 4.13 (0.78) |  | 4.59 (0.67) | 4.30 (0.66) | 5.99 (1.07) |
| Jeollabuk-do |  | 2.98 (0.47) | 2.98 (0.49) | 2.98 (0.71) |  | 3.06 (0.47) | 3.02 (0.50) | 3.25 (0.58) |
| Jeollanam-do |  | 2.80 (0.48) | 2.83 (0.52) | 2.75 (0.51) |  | 3.24 (0.51) | 2.96 (0.48) | 4.63 (0.92) |
| Gyeongsangbuk-do |  | 5.58 (0.72) | 5.39 (0.80) | 6.01 (0.84) |  | 5.79 (0.74) | 5.58 (0.72) | 6.85 (1.24) |
| Gyeongsangnam-do |  | 6.83 (0.95) | 6.51 (0.99) | 7.55 (1.20) |  | 5.73 (0.77) | 5.84 (0.81) | 5.16 (0.99) |
| Jeju-do |  | 1.82 (0.67) | 1.80 (0.72) | 1.87 (0.73) |  | 1.95 (0.96) | 2.02 (1.05) | 1.56 (0.57) |
| Town | .891 |  |  |  | .018 |  |  |  |
| Dong (city) |  | 80.62 (1.83) | 80.56 (1.96) | 80.77 (1.97) |  | 80.37 (1.81) | 81.12 (1.78) | 76.61 (2.68) |
| Eup, Myeon (rural) |  | 19.38 (1.83) | 19.44 (1.96) | 19.23 (1.97) |  | 19.63 (1.81) | 18.88 (1.78) | 23.39 (2.68) |
| Income | .054 |  |  |  | .225 |  |  |  |
| 1st quartile (low) |  | 25.78 (0.96) | 24.67 (1.14) | 28.25 (1.55) |  | 26.42 (0.85) | 26.06 (0.94) | 28.2 (1.82) |
| 2nd quartile (lower-middle) |  | 26.27 (0.89) | 26.03 (1.08) | 26.82 (1.35) |  | 25.37 (0.74) | 25.10 (0.82) | 26.71 (1.57) |
| 3rd quartile (upper-middle) |  | 23.33 (0.76) | 23.44 (0.96) | 23.09 (1.31) |  | 24.97 (0.73) | 25.05 (0.8) | 24.54 (1.46) |
| 4th quartile (high) |  | 24.62 (0.98) | 25.87 (1.15) | 21.84 (1.40) |  | 23.24 (0.84) | 23.78 (0.93) | 20.56 (1.47) |
| Education | <.001 |  |  |  | <.001 |  |  |  |
| Elementary school or less |  | 11.98 (0.60) | 10.57 (0.65) | 15.13 (1.08) |  | 25.5 (0.89) | 22.34 (0.90) | 41.21 (1.99) |
| Middle school |  | 10.32 (0.52) | 9.77 (0.64) | 11.52 (0.94) |  | 9.50 (0.44) | 9.55 (0.49) | 9.21 (0.97) |
| High school |  | 40.35 (1.06) | 42.02 (1.25) | 36.61 (1.66) |  | 37.45 (0.96) | 38.81 (1.09) | 30.67 (1.84) |
| University or higher |  | 37.36 (1.13) | 37.63 (1.30) | 36.74 (1.63) |  | 27.56 (0.89) | 29.3 (1.00) | 18.91 (1.53) |
| Occupation | 0.71 |  |  |  | <.001 |  |  |  |
| Managers, professionals and related workers |  | 17.91 (0.79) | 17.5 (0.92) | 18.81 (1.23) |  | 10.23 (0.51) | 11.21 (0.59) | 5.34 (0.85) |
| Clerks |  | 11.60 (0.59) | 11.96 (0.70) | 10.80 (0.93) |  | 6.39 (0.36) | 6.81 (0.41) | 4.31 (0.75) |
| Service workers and sale workers |  | 12.84 (0.66) | 13.1 (0.79) | 12.25 (1.02) |  | 14.55 (0.62) | 14.96 (0.63) | 12.50 (1.47) |
| Skilled agricultural, forestry and fishery workers |  | 7.71 (0.89) | 7.83 (0.97) | 7.45 (0.97) |  | 5.51 (0.66) | 5.21 (0.68) | 6.96 (1.00) |
| Craft, plant, machine operators and assemblers |  | 20.09 (0.82) | 20.05 (0.99) | 20.19 (1.35) |  | 2.30 (0.23) | 2.16 (0.23) | 3.02 (0.65) |
| Elementary occupations |  | 7.25 (0.49) | 7.50 (0.60) | 6.68 (0.85) |  | 8.40 (0.44) | 8.56 (0.48) | 7.56 (0.95) |
| Unemployed |  | 22.61 (0.91) | 22.07 (1.11) | 23.82 (1.34) |  | 52.62 (0.86) | 51.08 (0.91) | 60.30 (1.98) |
| Drinking | <.001 |  |  |  | <.001 |  |  |  |
| Not at all for the past one year |  | 13.01 (0.65) | 10.29 (0.64) | 19.08 (1.36) |  | 33.08 (0.84) | 31.05 (0.88) | 43.19 (2.03) |
| Less than once a month |  | 9.68 (0.61) | 9.07 (0.69) | 11.04 (1.03) |  | 26.72 (0.68) | 26.75 (0.76) | 26.55 (1.7) |
| Once a month |  | 9.23 (0.55) | 8.80 (0.65) | 10.18 (1.04) |  | 11.73 (0.49) | 11.63 (0.55) | 12.23 (1.13) |
| 2 to 4 times a month |  | 30.70 (0.87) | 30.10 (1.02) | 32.04 (1.50) |  | 19.48 (0.66) | 20.42 (0.74) | 14.82 (1.29) |
| 2 or 3 times a week |  | 24.23 (0.78) | 26.96 (0.99) | 18.16 (1.20) |  | 6.94 (0.44) | 7.87 (0.51) | 2.29 (0.53) |
| 4 or more times a week |  | 13.14 (0.62) | 14.78 (0.79) | 9.49 (0.93) |  | 2.05 (0.22) | 2.27 (0.27) | 0.93 (0.35) |
| Smoking | .401 |  |  |  | .423 |  |  |  |
| Smoking |  | 46.40 (0.99) | 45.66 (1.22) | 48.05 (1.60) |  | 6.58 (0.42) | 6.41 (0.49) | 7.41 (0.98) |
| Quit smoking |  | 33.40 (0.87) | 33.58 (1.08) | 32.99 (1.50) |  | 6.39 (0.41) | 6.58 (0.46) | 5.49 (0.92) |
| Never smoked |  | 20.20 (0.76) | 20.75 (0.95) | 18.96 (1.29) |  | 87.03 (0.58) | 87.01 (0.67) | 87.11 (1.25) |
| Stress | .654 |  |  |  | .017 |  |  |  |
| Extremely |  | 4.10 (0.34) | 4.38 (0.43) | 3.48 (0.55) |  | 5.26 (0.34) | 5.14 (0.35) | 5.88 (0.94) |
| Very |  | 22.36 (0.79) | 22.52 (0.98) | 22.02 (1.38) |  | 26.32 (0.72) | 26.48 (0.78) | 25.51 (1.66) |
| Slightly |  | 59.50 (0.88) | 59.22 (1.10) | 60.12 (1.49) |  | 56.18 (0.75) | 56.83 (0.86) | 52.90 (2.04) |
| Rarely |  | 14.04 (0.59) | 13.88 (0.74) | 14.38 (1.05) |  | 12.24 (0.48) | 11.54 (0.51) | 15.71 (1.30) |
| Exercise | .183 |  |  |  | .030 |  |  |  |
| Not at all |  | 55.80 (0.97) | 54.55 (1.18) | 58.60 (1.71) |  | 74.19 (0.80) | 73.4 (0.84) | 78.1 (1.67) |
| Once a week |  | 15.66 (0.75) | 16.07 (0.94) | 14.74 (1.17) |  | 6.56 (0.39) | 6.89 (0.44) | 4.90 (0.78) |
| 2 times a week |  | 9.72 (0.54) | 9.93 (0.67) | 9.26 (0.95) |  | 5.24 (0.34) | 5.46 (0.37) | 4.14 (0.67) |
| 3 times a week |  | 7.26 (0.45) | 7.50 (0.57) | 6.71 (0.81) |  | 5.74 (0.41) | 5.71 (0.44) | 5.89 (0.99) |
| 4 times a week |  | 3.19 (0.33) | 2.92 (0.37) | 3.78 (0.71) |  | 2.23 (0.26) | 2.36 (0.30) | 1.57 (0.48) |
| 5 times a week |  | 3.49 (0.34) | 3.89 (0.45) | 2.6 (0.46) |  | 2.78 (0.25) | 3.06 (0.28) | 1.42 (0.45) |
| 6 times a week |  | 2.12 (0.27) | 2.41 (0.36) | 1.46 (0.39) |  | 0.97 (0.14) | 0.96 (0.16) | 1.01 (0.41) |
| Every day |  | 2.76 (0.30) | 2.73 (0.33) | 2.83 (0.59) |  | 2.29 (0.28) | 2.16 (0.29) | 2.96 (0.69) |

BMI: body mass index; SBP: systolic blood pressure; DBP: diastolic blood pressure; AST: aspartate aminotransferase; ALT: alanine aminotransferase. Continuous variables are represented as the mean ± standard error (SE) by complex-samples general linear models, and categorical variables are represented as the percentage (SE) by Rao-Scott chi-square tests. All statistical analyses were conducted using weight, cluster and stratification parameters to consider complex-sample survey data.

**Supplementary Table 6.** Basic characteristics of hypertriglyceridemia and normal groups.

| Variables | Men |  |  |  | Women |  |  |  |
| --- | --- | --- | --- | --- | --- | --- | --- | --- |
|  | P-value |  | Normal | hypertriglyceridemia | P-value |  | Normal | hypertriglyceridemia |
| Number of subjects |  |  | 3,441 | 992 |  |  | 5,724 | 633 |
| Age (years) | <.001 |  | 42.95 ± 0.37 | 46.52 ± 0.49 | <.001 |  | 44.48 ± 0.33 | 55.37 ± 0.86 |
| BMI (kg/m^2^) | <.001 |  | 23.67 ± 0.07 | 25.28 ± 0.10 | <.001 |  | 23.12 ± 0.06 | 25.23 ± 0.19 |
| Waist circumference (cm) | <.001 |  | 82.64 ± 0.21 | 88.17 ± 0.29 | <.001 |  | 77.31 ± 0.20 | 84.37 ± 0.47 |
| Waist-to-height ratio | <.001 |  | 0.48 ± 0.001 | 0.52 ± 0.002 | <.001 |  | 0.49 ± 0.001 | 0.55 ± 0.003 |
| SBP (mmHg) | <.001 |  | 120.07 ± 0.31 | 126.48 ± 0.65 | <.001 |  | 114.86 ± 0.34 | 126.19 ± 0.85 |
| DBP (mmHg) | <.001 |  | 79.62 ± 0.24 | 84.82 ± 0.47 | <.001 |  | 74.02 ± 0.20 | 78.83 ± 0.53 |
| Pulse rate (beats per 15 seconds) | <.001 |  | 17.48 ± 0.05 | 17.89 ± 0.09 | .421 |  | 17.91 ± 0.05 | 18.00 ± 0.11 |
| Hemoglobin (mg/dl) | <.001 |  | 15.26 ± 0.03 | 15.58 ± 0.04 | <.001 |  | 12.94 ± 0.02 | 13.26 ± 0.05 |
| Cholesterol (mg/dl) | <.001 |  | 182.42 ± 0.71 | 204.93 ± 1.60 | <.001 |  | 184.65 ± 0.60 | 211.51 ± 1.85 |
| Triglyceride (mg/dl) | <.001 |  | 107.80 ± 0.85 | 328.11 ± 7.43 | <.001 |  | 92.74 ± 0.72 | 286.32 ± 4.88 |
| Glucose (mg/dl) | <.001 |  | 95.82 ± 0.45 | 104.75 ± 0.99 | <.001 |  | 93.17 ± 0.28 | 106.55 ± 1.61 |
| AST (IU/L) | <.001 |  | 23.55 ± 0.27 | 29.85 ± 0.77 | <.001 |  | 19.53 ± 0.13 | 23.17 ± 0.49 |
| ALT (IU/L) | <.001 |  | 24.95 ± 0.38 | 35.56 ± 0.90 | <.001 |  | 16.67 ± 0.20 | 23.11 ± 0.67 |
| Creatinine (mg/dl) | .126 |  | 0.96 ± 0.01 | 0.95 ± 0.01 | .035 |  | 0.70 ± 0.002 | 0.72 ± 0.01 |
| Trunk fat mass (kg) | <.001 |  | 8.14 ± 0.09 | 10.16 ± 0.12 | <.001 |  | 9.49 ± 0.07 | 11.83 ± 0.18 |
| Percent trunk fat mass (%) | <.001 |  | 23.53 ± 0.18 | 27.61 ± 0.22 | <.001 |  | 32.78 ± 0.16 | 37.45 ± 0.30 |
| Whole-body total fat mass (kg) | <.001 |  | 15.16 ± 0.15 | 17.78 ± 0.19 | <.001 |  | 19.06 ± 0.11 | 21.41 ± 0.32 |
| Percent whole-body total fat mass (%) | <.001 |  | 21.57 ± 0.16 | 24.32 ± 0.19 | <.001 |  | 32.98 ± 0.13 | 35.30 ± 0.28 |
| Mean sleep duration (hours) | .705 |  | 6.89 ± 0.03 | 6.91 ± 0.04 | .502 |  | 6.88 ± 0.02 | 6.82 ± 0.08 |
| Region (city) | .017 |  |  |  | .587 |  |  |  |
| Seoul |  | 21.58 (1.10) | 22.57 (1.20) | 17.99 (1.70) |  | 20.8 (0.91) | 20.68 (0.93) | 22.05 (2.50) |
| Busan |  | 7.89 (0.74) | 7.50 (0.78) | 9.30 (1.36) |  | 7.82 (0.84) | 7.94 (0.91) | 6.62 (1.39) |
| Daegu |  | 5.15 (0.71) | 4.95 (0.73) | 5.86 (1.12) |  | 5.11 (0.70) | 5.28 (0.70) | 3.46 (0.91) |
| Incheon |  | 5.30 (0.58) | 5.21 (0.61) | 5.65 (0.92) |  | 5.15 (0.52) | 5.21 (0.53) | 4.49 (1.10) |
| Gwangju |  | 2.27 (0.69) | 2.55 (0.81) | 1.24 (0.41) |  | 2.21 (0.60) | 2.24 (0.62) | 1.90 (0.57) |
| Daejeon |  | 3.18 (0.68) | 3.39 (0.8) | 2.45 (0.78) |  | 3.36 (0.68) | 3.36 (0.67) | 3.40 (1.14) |
| Ulsan |  | 2.11 (0.64) | 1.71 (0.54) | 3.56 (1.24) |  | 2.55 (0.75) | 2.50 (0.74) | 3.05 (1.22) |
| Gyeonggi-do |  | 22.85 (1.11) | 22.61 (1.15) | 23.73 (1.8) |  | 22.98 (0.97) | 22.96 (1.01) | 23.21 (2.33) |
| Gangwon-do |  | 2.34 (0.39) | 2.46 (0.44) | 1.90 (0.48) |  | 2.32 (0.5) | 2.31 (0.53) | 2.42 (0.78) |
| Chungcheongbuk-do |  | 3.36 (0.61) | 3.21 (0.60) | 3.88 (0.85) |  | 3.34 (0.57) | 3.21 (0.55) | 4.67 (1.52) |
| Chungcheongnam-do |  | 3.95 (0.66) | 3.76 (0.77) | 4.63 (0.87) |  | 4.59 (0.67) | 4.51 (0.68) | 5.37 (1.25) |
| Jeollabuk-do |  | 2.98 (0.47) | 2.75 (0.43) | 3.81 (0.99) |  | 3.06 (0.47) | 3.01 (0.47) | 3.49 (1.08) |
| Jeollanam-do |  | 2.80 (0.48) | 2.86 (0.49) | 2.61 (0.67) |  | 3.24 (0.51) | 3.21 (0.51) | 3.54 (1.01) |
| Gyeongsangbuk-do |  | 5.58 (0.72) | 5.85 (0.84) | 4.64 (0.79) |  | 5.79 (0.74) | 5.67 (0.73) | 7.00 (1.20) |
| Gyeongsangnam-do |  | 6.83 (0.95) | 6.83 (0.96) | 6.84 (1.18) |  | 5.73 (0.77) | 5.91 (0.80) | 3.92 (1.10) |
| Jeju-do |  | 1.82 (0.67) | 1.80 (0.67) | 1.91 (0.75) |  | 1.95 (0.96) | 2.00 (0.96) | 1.41 (0.90) |
| Town | .131 |  |  |  | .009 |  |  |  |
| Dong (city) |  | 80.62 (1.83) | 81.18 (1.83) | 78.60 (2.38) |  | 80.37 (1.81) | 80.87 (1.78) | 75.42 (2.99) |
| Eup, Myeon (rural) |  | 19.38 (1.83) | 18.82 (1.83) | 21.40 (2.38) |  | 19.63 (1.81) | 19.13 (1.78) | 24.58 (2.99) |
| Income | .281 |  |  |  | .285 |  |  |  |
| 1st quartile (low) |  | 25.78 (0.96) | 26.08 (1.04) | 24.69 (1.81) |  | 26.42 (0.85) | 26.10 (0.88) | 29.57 (2.56) |
| 2nd quartile (lower-middle) |  | 26.27 (0.89) | 25.47 (0.94) | 29.18 (1.91) |  | 25.37 (0.74) | 25.39 (0.80) | 25.15 (2.16) |
| 3rd quartile (upper-middle) |  | 23.33 (0.76) | 23.40 (0.87) | 23.08 (1.68) |  | 24.97 (0.73) | 24.91 (0.75) | 25.51 (2.09) |
| 4th quartile (high) |  | 24.62 (0.98) | 25.06 (1.05) | 23.04 (1.80) |  | 23.24 (0.84) | 23.59 (0.89) | 19.77 (1.98) |
| Education | <.001 |  |  |  | <.001 |  |  |  |
| Elementary school or less |  | 11.98 (0.60) | 12.06 (0.68) | 11.70 (1.13) |  | 25.5 (0.89) | 23.17 (0.85) | 48.66 (2.67) |
| Middle school |  | 10.32 (0.52) | 9.11 (0.54) | 14.65 (1.28) |  | 9.50 (0.44) | 9.25 (0.46) | 11.96 (1.52) |
| High school |  | 40.35 (1.06) | 41.37 (1.19) | 36.65 (1.83) |  | 37.45 (0.96) | 38.45 (1.00) | 27.47 (2.35) |
| University or higher |  | 37.36 (1.13) | 37.45 (1.21) | 37.00 (2.00) |  | 27.56 (0.89) | 29.13 (0.95) | 11.91 (1.76) |
| Occupation | .001 |  |  |  | <.001 |  |  |  |
| Managers, professionals and related workers |  | 17.91 (0.79) | 17.74 (0.88) | 18.52 (1.64) |  | 10.23 (0.51) | 10.89 (0.56) | 3.67 (1.03) |
| Clerks |  | 11.60 (0.59) | 11.42 (0.65) | 12.24 (1.27) |  | 6.39 (0.36) | 6.78 (0.39) | 2.54 (0.78) |
| Service workers and sale workers |  | 12.84 (0.66) | 13.48 (0.77) | 10.51 (1.07) |  | 14.55 (0.62) | 14.76 (0.62) | 12.49 (1.88) |
| Skilled agricultural, forestry and fishery workers |  | 7.71 (0.89) | 7.70 (0.95) | 7.76 (1.17) |  | 5.51 (0.66) | 5.15 (0.66) | 9.04 (1.51) |
| Craft, plant, machine operators and assemblers |  | 20.09 (0.82) | 18.59 (0.90) | 25.52 (1.8) |  | 2.30 (0.23) | 2.31 (0.24) | 2.20 (0.74) |
| Elementary occupations |  | 7.25 (0.49) | 7.87 (0.59) | 5.00 (0.87) |  | 8.40 (0.44) | 8.28 (0.46) | 9.59 (1.51) |
| Unemployed |  | 22.61 (0.91) | 23.21 (1.04) | 20.44 (1.54) |  | 52.62 (0.86) | 51.83 (0.88) | 60.47 (2.51) |
| Drinking | <.001 |  |  |  | <.001 |  |  |  |
| Not at all for the past one year |  | 13.01 (0.65) | 13.83 (0.77) | 10.06 (1.10) |  | 33.08 (0.84) | 32.04 (0.86) | 43.40 (2.45) |
| Less than once a month |  | 9.68 (0.61) | 10.81 (0.71) | 5.61 (0.85) |  | 26.72 (0.68) | 26.80 (0.72) | 25.90 (2.15) |
| Once a month |  | 9.23 (0.55) | 10.22 (0.64) | 5.64 (0.85) |  | 11.73 (0.49) | 12.14 (0.53) | 7.66 (1.20) |
| 2 to 4 times a month |  | 30.70 (0.87) | 31.54 (0.96) | 27.70 (1.78) |  | 19.48 (0.66) | 19.90 (0.72) | 15.32 (1.73) |
| 2 or 3 times a week |  | 24.23 (0.78) | 21.75 (0.83) | 33.20 (1.93) |  | 6.94 (0.44) | 7.10 (0.45) | 5.30 (1.30) |
| 4 or more times a week |  | 13.14 (0.62) | 11.85 (0.62) | 17.79 (1.54) |  | 2.05 (0.22) | 2.01 (0.24) | 2.43 (0.72) |
| Smoking | <.001 |  |  |  | .019 |  |  |  |
| Smoking |  | 46.4 (0.99) | 43.85 (1.08) | 55.61 (1.89) |  | 6.58 (0.42) | 6.22 (0.45) | 10.11 (1.47) |
| Quit smoking |  | 33.4 (0.87) | 33.58 (0.94) | 32.73 (1.81) |  | 6.39 (0.41) | 6.55 (0.44) | 4.80 (1.31) |
| Never smoked |  | 20.2 (0.76) | 22.57 (0.93) | 11.66 (1.15) |  | 87.03 (0.58) | 87.22 (0.63) | 85.09 (1.90) |
| Stress | .543 |  |  |  | .001 |  |  |  |
| Extremely |  | 4.10 (0.34) | 4.21 (0.38) | 3.70 (0.68) |  | 5.26 (0.34) | 4.93 (0.32) | 8.61 (1.67) |
| Very |  | 22.36 (0.79) | 21.95 (0.90) | 23.86 (1.79) |  | 26.32 (0.72) | 26.66 (0.74) | 22.92 (2.08) |
| Slightly |  | 59.50 (0.88) | 60.03 (0.96) | 57.58 (1.94) |  | 56.18 (0.75) | 56.56 (0.78) | 52.32 (2.46) |
| Rarely |  | 14.04 (0.59) | 13.81 (0.63) | 14.86 (1.43) |  | 12.24 (0.48) | 11.85 (0.50) | 16.15 (1.75) |
| Exercise | .666 |  |  |  | .111 |  |  |  |
| Not at all |  | 55.8 (0.97) | 56.01 (1.05) | 55.07 (2.00) |  | 74.19 (0.80) | 73.61 (0.83) | 79.94 (2.10) |
| Once a week |  | 15.66 (0.75) | 15.33 (0.79) | 16.84 (1.59) |  | 6.56 (0.39) | 6.72 (0.41) | 4.99 (1.16) |
| 2 times a week |  | 9.72 (0.54) | 9.48 (0.62) | 10.59 (1.15) |  | 5.24 (0.34) | 5.37 (0.36) | 3.95 (0.82) |
| 3 times a week |  | 7.26 (0.45) | 7.20 (0.50) | 7.47 (1.03) |  | 5.74 (0.41) | 5.83 (0.44) | 4.83 (1.15) |
| 4 times a week |  | 3.19 (0.33) | 3.35 (0.39) | 2.58 (0.58) |  | 2.23 (0.26) | 2.25 (0.27) | 2.04 (0.66) |
| 5 times a week |  | 3.49 (0.34) | 3.52 (0.40) | 3.39 (0.67) |  | 2.78 (0.25) | 2.96 (0.27) | 0.98 (0.52) |
| 6 times a week |  | 2.12 (0.27) | 2.10 (0.30) | 2.17 (0.65) |  | 0.97 (0.14) | 1.02 (0.16) | 0.48 (0.30) |
| Every day |  | 2.76 (0.30) | 3.01 (0.35) | 1.88 (0.50) |  | 2.29 (0.28) | 2.25 (0.28) | 2.78 (1.03) |

BMI: body mass index; SBP: systolic blood pressure; DBP: diastolic blood pressure; AST: aspartate aminotransferase; ALT: alanine aminotransferase. Continuous variables are represented as the mean ± standard error (SE) by complex-samples general linear models, and categorical variables are represented as the percentage (SE) by Rao-Scott chi-square tests. All statistical analyses were conducted using weight, cluster and stratification parameters to consider complex-sample survey data.
